# Intrinsic Disordered Network in Multiferroic YMnO_3_ Single Crystals for In‐Materio Physical Reservoir Computing Through Tuneable Domain‐Wall Structure

**DOI:** 10.1002/smll.202506397

**Published:** 2025-09-12

**Authors:** Muzhen Xu, Kyoka Furuta, Ahmet Karacali, Yuki Umezaki, Alif Syafiq Kamarol Zaman, Yuki Usami, Hirofumi Tanaka, Yoichi Horibe

**Affiliations:** ^1^ Research Center for Neuromorphic AI Hardware Kyushu Institute of Technology 2–4 Hibikino, Wakamatsu Kitakyushu 808–0196 Japan; ^2^ Department of Materials Science and Engineering Kyushu Institute of Technology 1–1 Sensui, Tobata Kitakyushu 804–8550 Japan; ^3^ Department of Human Intelligence Systems Kyushu Institute of Technology 2–4 Hibikino, Wakamatsu Kitakyushu 808–0196 Japan

**Keywords:** domain structure, in‐materio reservoir computing, physical reservoir computing, YMnO_3_, yttrium manganese oxide

## Abstract

Physical reservoir computing (PRC) is an innovative computational paradigm that leverages intrinsic nonlinearity of physical systems to efficiently perform complex tasks. It is discovered that the intrinsically disordered domain structure in multiferroic YMnO_3_ provides significant nonlinearity, making it a promising candidate for robust PRC with tuneability and functionality at high temperatures. This work explores the potential of YMnO_3_ single crystals for PRC. PRC performance of YMnO_3_ is systematically evaluated by analysing its nonlinear responses, phase shifts, and high dimensionality through benchmark tasks such as waveform generation (WG), memory capacity (MC), and second‐order nonlinear autoregressive moving average (NARMA2) time‐series prediction. This results demonstrate that YMnO_3_ single crystals exhibit superior performance in these tasks, achieving high accuracy and low power consumption (≈1.77 µW and ≈0.02 nW/domain). These crystals also performed well in practical application of low‐power speech recognition. These findings establish YMnO_3_ as a viable platform for next‐generation PRC technologies, addressing critical challenges in the field.

## Graphical abstract

Intrinsically disordered cloverleaf domain structures in multiferroic YMnO_3_ single crystals enable highly nonlinear, thermally stable, and energy‐efficient physical reservoir computing. This system performs waveform generation, time‐series forecasting, and voice recognition at microwatt‐level power, leveraging the anisotropic domain‐wall topology to transform input signals into high‐dimensional output dynamics.

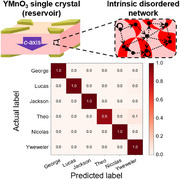

## 1 Introduction

The escalating demand for intelligent, adaptive, and sustainable computing paradigms has catalyzed intense interest in brain‐inspired hardware systems that transcend the limitations of conventional von Neumann architectures.^[^ ^1^, ^2^ ^]^ In particular, neuromorphic engineering—an interdisciplinary field converging neuroscience, materials science, and electronics—has emerged as a vanguard for enabling perception, learning, and decision‐making at the edge, with minimal energy expenditure. As artificial intelligence (AI) continues to migrate toward distributed, low‐power platforms for real‐time processing of complex temporal signals—such as voice, motion, and biosignals—there is a critical need for hardware that can emulate the cognitive functionalities of the human brain, including memory retention, nonlinear information transformation, and parallelism, all under stringent power and space constraints.^[^ ^3^, ^4^ ^]^

**Figure 1:**
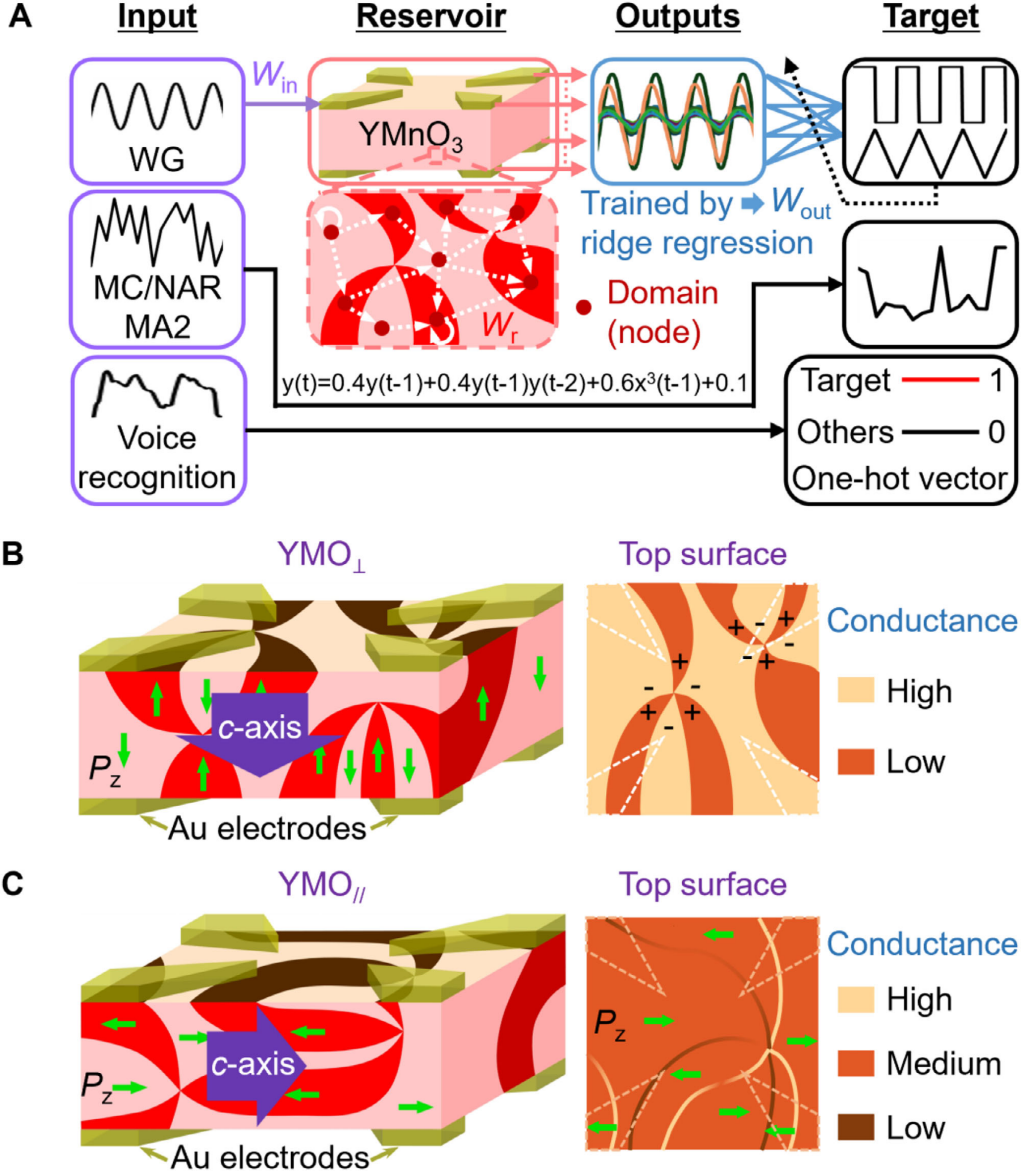
YMnO_3_ single crystal‐based in‐materio PRC device. A) Schematic of PRC system consists of three parts: input layer, reservoir, and output layer. Input weights (*W*_in_) and weights of recurrent connections inside reservoir (*W*_r_) are fixed whereas output weights (*W*_out_) are flexible and trained to fit target. B, C) Schematic of (left) YMnO_3_ single crystal (YMO_⊥_, (D), YMO_//_, (C)) sandwiched by top and bottom electrode arrays and (right) conductance distribution on their corresponding top surfaces. YMnO_3_ single crystal inherently shows cloverleaf‐like domain configurations. Green arrows indicate domains’ polarization directions (*P*_z_). Purple arrow indicates crystallographic *c*‐axis. + and – symbols indicate the upward and downward polarization directions, respectively. Dotted lines indicate the position of Au electrodes.

Among emerging approaches, physical reservoir computing (PRC) has garnered significant traction as a minimalist yet potent neuromorphic paradigm that exploits the rich internal dynamics of physical systems to process spatiotemporal data without iterative training of internal states.^[^ ^3^, ^4^, ^5^ ^]^ Unlike conventional deep neural networks that require extensive optimization and high computational cost, PRC utilizes a fixed nonlinear medium—or “reservoir”—to transform input streams into high‐dimensional representations, which are linearly combined at the output (**Figure** **1**A). This architecture mirrors the echo state dynamics of cortical microcircuits, where transient memory and nonlinear integration are naturally encoded in the system's intrinsic physical evolution.^[^ ^6^ ^]^ Various material platforms—ranging from optical resonators and spintronic networks to electrochemical and memristive systems—have demonstrated proof‐of‐concept PRC devices, showcasing applications in signal classification, time‐series forecasting, and pattern recognition.^[^ ^7^, ^8^, ^9^, ^10^, ^11^, ^12^, ^13^, ^14^, ^15^ ^]^ While most PRC research has focused on optical, electrochemical, or memristive systems, the application of multiferroic materials in this field remains largely unexplored. Recently, Sun et al., using a Pt/Co/Gd‐PMN‑PT stack^[^ ^16^ ^]^ (skyrmion‑enhanced, strain‑mediated) and a PMN‑PT substrate,^[^ ^17^ ^]^ demonstrated high accuracy in waveform classification and Mackey–Glass prediction. These multiferroic heterostructures offer fast response, multi‐modal tuning, and low power consumption, making them a promising platform for future PRC applications. Despite these advances, most PRC materials and systems remain constrained by fundamental limitations—particularly thermal instability, material degradation, and narrow operational temperature ranges—which obstruct their seamless integration with complementary metal‐oxide‐semiconductor (CMOS) technologies. CMOS back‐end‐of‐line (BEOL) processes typically involve annealing steps at temperatures up to 400–500 °C.^[^ ^18^ ^]^ The lack of materials that combine strong nonlinearity, stable dynamic behavior, and resilience under harsh conditions continues to hinder the realization of scalable and reliable physical computing architectures.

In this context, multiferroic yttrium manganese oxide (YMnO_3_) single crystal with intriguing domain/domain wall structures presents a compelling alternative. Domains, the spatial distributions of orientational order parameters, are commonly found in various materials, including magnetic,^[^ ^19^ ^]^ ferroelectric,^[^ ^20^ ^]^ and crystallographic^[^ ^21^ ^]^ systems. These domains are often viewed as undesirable due to their nonlinear behaviour, which complicates the material's overall performance. However, domain‐induced nonlinearity is essential, though largely unexplored, in the context of PRC. The unique domain structures were found and well‐studied in multiferroic yttrium manganese oxide (YMnO_3_).^[^ ^22^, ^23^ ^]^ The cloverleaf‐like arrangement of ferroelectric and antiphase domains in YMnO_3_ gives rise to intriguing physical properties, such as inhomogeneous conduction linked to domain polarization and a network of conducting and insulating domain walls, which depends on the relative polarisation relationships.^[^ ^24^ ^]^ These domain‐related properties exhibit several key functionalities ideally suited for PRC applications. First, the material displays highly nonlinear current–voltage (*I*–*V*) characteristics resulting from space‐charge‐limited conduction (SCLC) mechanisms, a crucial factor for achieving the nonlinearity and high dimensionality needed in physical reservoirs. Additionally, YMnO_3_ exhibits intrinsic phase delays and shifts in its dynamic response to external inputs, which emulate the fading memory capabilities of abstract reservoir models. The complicated configurations of ferroelectric domains in YMnO_3_ further enhance its high dimensionality by providing a vast configuration space for accessible domain states. Importantly, YMnO_3_ is a fully solid, crystalline material with a self‐formed domain‐wall structure that remains stable up to 800 °C, offering significant advantages in terms of stability, scalability, integration, and environmental robustness compared to other physical reservoir materials. With its unique combination of nonlinear *I*–*V* behaviour, phase dynamics, high dimensionality, and solid‐state nature, YMnO_3_ single crystals present a promising platform for developing ultra‐compact, energy‐efficient PRC devices for applications such as waveform generation (WG), time‐series forecasting, and speech recognition.

Motivated by the unique properties of YMnO_3_, which are well‐suited for PRC, we conducted a comprehensive evaluation of the potential of YMnO_3_ single crystals as physical reservoirs for computing tasks. The primary objectives of this study are threefold: 1) To systematically characterise the PRC performance of YMnO_3_ crystals by examining their nonlinear responses, phase shifts, high dimensionality capabilities, and performance on benchmark tasks such as WG, memory capacity (MC), and second‐order nonlinear autoregressive moving average (NARMA2) time‐series prediction problems. 2) To optimise the PRC capabilities of YMnO_3_ by exploring the effects of device configuration (perpendicular versus parallel to the c‐axis, same side versus opposite‐side electrodes), operating conditions such as input voltage amplitudes, and the influence of domain structure. 3) To demonstrate practical applications that exploit the unique properties of YMnO_3_ reservoirs, such as low‐power speech recognition. Our findings position YMnO_3_ as a next‐generation reservoir material that meets the multifaceted demands of green AI, edge computing, and scalable neuromorphic hardware.

## 2 Results and Discussion

### 2.1 YMnO_3_ Crystal and Device Configurations

Multiferroic YMnO_3_ exhibits a distinctive improper ferroelectricity characterized by interlocked structural and polar domain walls, forming a topologically protected cloverleaf‐like network (Figure 1B,C). Cloverleaf patterns were clearly observed by transmission electron microscopy (TEM) (Figure S1A, Supporting Information) and consistent with the observations of Choi et al.^[^ ^22^ ^]^ and Meier et al.^[^ ^23^ ^]^ As thoroughly analyzed by Choi et al.,^[^ ^22^ ^]^ these characteristic patterns indicate that each vortex core in YMnO_3_ binds six alternating trimerization (antiphase) and polarization domains. These domain/domain walls are not merely passive bulk/interfaces but act as active electronic elements exhibiting ferroelectric polarization direction‐dependent conduction. Figure 1B,C present schematic illustrations derived from the c‐AFM measurements reported by Choi et al.^[^ ^22^ ^]^ and Meier et al.,^[^ ^23^ ^]^ respectively, highlighting the polarization‐dependent conduction at domains and domain walls in YMnO_3_. In the YMO_⊥_ configuration (Figure 1B), the polarization direction (*P*z) is perpendicular (90° or 270°) to the crystal surface. As demonstrated by Choi et al.,^[^ ^22^ ^]^ the domains are more conductive at downward polarizations (−*P*z) than those at upward polarizations (+*P*z) (exemplified in the right panel of Figure 1B). In contrast, in the YMO_//_ configuration (Figure 1C), *P*z is parallel (0° or 180°) to the crystal surface. Its electrical conductance at the domain walls is demonstrated to depend on a relative polarization relationship. As exemplified in the right panel of Figure 1C and consistent with the observations of Meier et al.,^[^ ^23^ ^]^ tail‐to‐tail domain walls are conductive and head‐to‐head domain walls are insulating. This spatial diversity of responses—stemming from microscopic domain architectures—creates a naturally high‐dimensional input‐output mapping, which is highly desirable for physical reservoir computing (PRC).

To systematically interrogate the influence of crystallographic orientation and domain wall alignment, we designed four distinct electrode configurations across YMnO_3_ crystallites with well‐defined orientations: YMO_⊥,s_: electrodes on the same surface, probing in‐plane transport perpendicular to the c‐axis, YMO_//,s_: electrodes on the same surface, probing in‐plane transport parallel to the c‐axis, YMO_⊥,o_: electrodes on opposite surfaces, probing vertical transport across domains perpendicular to the c‐axis, YMO_//,o_: electrodes on opposite surfaces, probing vertical transport across domains parallel to the c‐axis. To obtain these device configurations, each crystallite is sandwiched between two arrays of 16 electrodes, placed on both the front and back sides (Figure S1B, C, Supporting Information). Figure S1D (Supporting Information) shows the spider‐like design of the 16 electrodes, with gaps between neighbouring electrodes and opposite‐side electrodes measuring approximately 13 µm and 130 µm, respectively. One electrode was designated for applying the input voltage signal, while the remaining 15 electrodes were used to read the output responses. This configuration allows us to explore 15 parallel reservoir readouts from a single input. Moreover, the spider‐like design creates varying distances between the input and each output electrode, introducing spatial diversity in signal propagation paths. This results in a richer variety of nonlinear and delayed responses across the device, which is crucial for enhancing the computational capability of the reservoir system.

Together, these four configurations allow for a comprehensive dissection of anisotropic carrier transport behavior governed by the interplay of domain topology, crystallographic orientation, and electrode placement. By mapping the relationship between conduction pathways and device geometry, we establish a foundation for tuning the nonlinear response, memory characteristics, and computational dimensionality of YMnO_3_‐based in‐materio physical reservoir systems.

### 2.2 Nonlinearity, Phase Shift, and High Dimensionality

**Figure 2:**
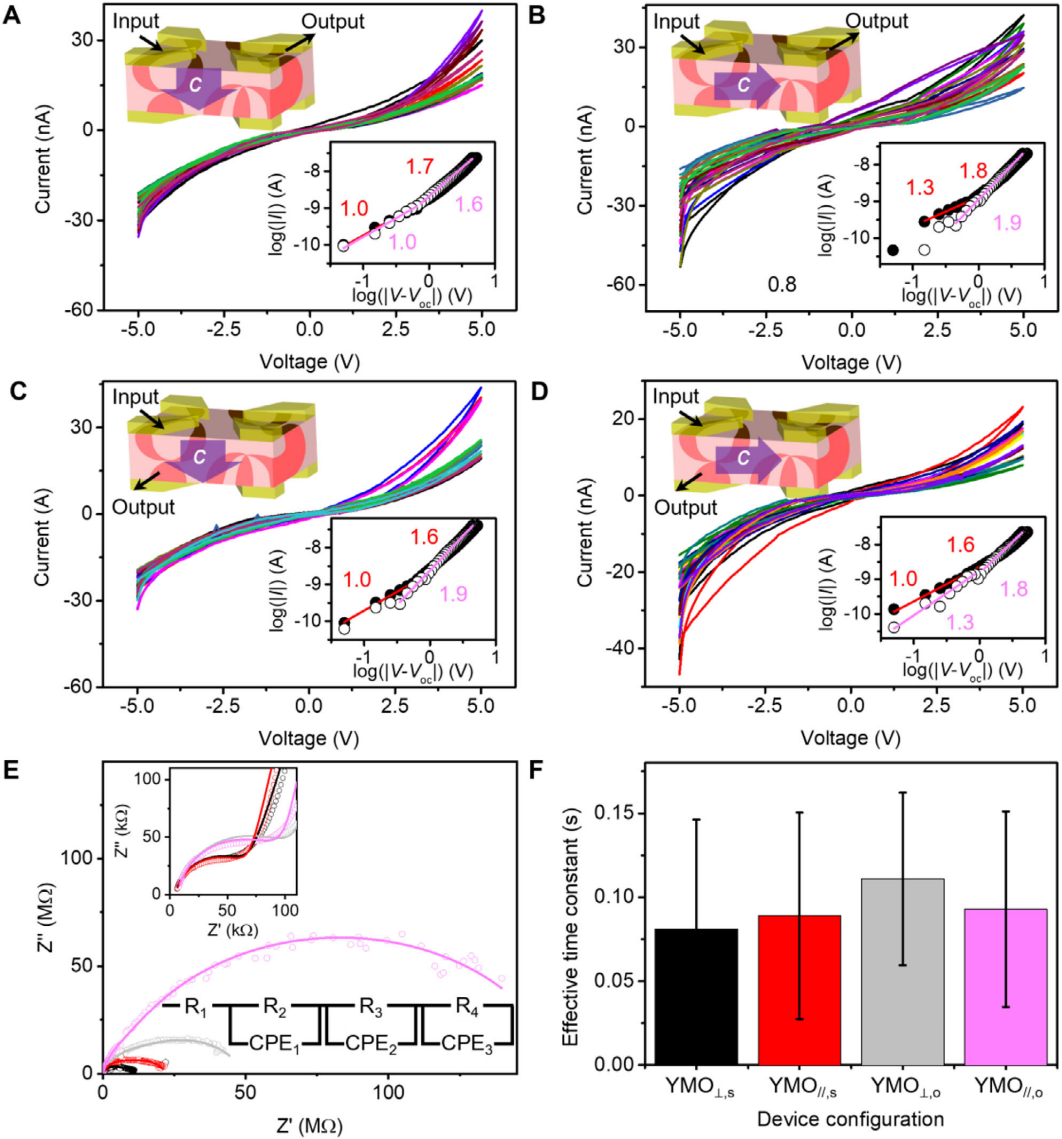
Nonlinearity and electrochemical impedance spectrum analysis across all device configurations. A–D) *I*–*V* output signals were measured for (A) YMO_⊥,s_: 15 signals, (B) YMO_//,s_: 15 signals, (C) YMO_⊥,o_: 16 signals, and (D) YMO_//,o_: 16 signals. Each top‐left inset shows corresponding schematic of device configuration. Each bottom‐right inset shows two corresponding log‐log scale *I*–*V* plots of output 1 (dot: forward scan, circle: backward scan). Each plot is linearly fitted to extract slope parameters, colour‐coded, and labelled accordingly. E) Cole‐Cole plots of output 1 measured on YMO_⊥,s_ (black), YMO_//,s_ (red), YMO_⊥,o_ (light grey), and YMO_//,o_ (light magenta), respectively. Top inset: Equivalent circuit diagram. Bottom inset: magnified high‐frequency region showing small semicircles. F) Average effective time constants calculated from outputs 1, 4, and 8 across all device configurations.

The *I*–*V* measurements revealed several key features that underpin the PRC capabilities of the YMnO_3_ crystals. Across all device configurations (YMO_⊥,s_, YMO_//,s_, YMO_⊥,o_, and YMO_//,o_), the *I*–*V* curves, shown in **Figure** **2A–D**, exhibited pronounced nonlinear characteristics—a crucial requirement for achieving the high dimensionality and nonlinear mapping necessary for PRC. A detailed analysis of the *I*–*V* curves on a log‐log scale (Figure 2A–D, bottom‐right insets) indicated that the current (*I*) was proportional to the voltage (*V*) raised to the power of *α* (I ∝ *V* ^α^). The values of *α*, indicated near the corresponding fitting lines, increased from approximately 1 to 2 as *V* increased, signifying a transition from ohmic to SCLC mechanisms, and reflecting an increase in nonlinearity with higher *V*. The nonlinearity across all device configurations was comparable, despite significant differences in electrode spacing for the same side (13–130 µm) and opposite side (1.5 mm) measurements, as well as differences in polarization direction for YMO_⊥_ and YMO_//_. It is possibly because the electrical nonlinearity is an intrinsic bulk property and thus remains consistent across different measurement geometries and polarization orientations. Consequently, the nonlinear characteristics crucial for PRC functionality are fundamentally independent of the external electrode configuration. This independence offers significant advantages for device architecture in PRC. It enables flexible electrode design, robust manufacturing tolerance, and scalability to large device arrays, facilitating integration into practical on‐chip computing systems.

Electrochemical impedance spectroscopy (EIS) measurements were conducted to investigate the charge transport and relaxation processes within the YMnO_3_ crystals, which are critical for optimising their performance in PRC applications. Figure 2E shows the EIS results for output 1 across all device configurations, revealing a non‐ideal semicircle in the Nyquist plots. This non‐ideal semicircle suggests the presence of constant phase elements (CPE) in parallel with resistors (R), forming (R‐CPE) circuits. These circuits may arise from factors such as surface roughness, leakage capacitance, and the non‐uniform distribution between domain walls and electrodes.^[^ ^25^, ^26^ ^]^ A closer examination of the high‐frequency region (Figure 2E, top‐left inset) reveals multiple semicircles in the Cole‐Cole plots, indicating distinct charge transport and relaxation processes. These findings suggest contributions from various mechanisms, including bulk resistance, domain, domain wall, and electrode/crystal interface effects.^[^ ^27^ ^]^ To model these processes, an equivalent circuit consisting of a resistor and three R‐CPE components connected in series was used (Figure 2E, bottom inset). This model effectively captured the complex impedance behaviour observed in the measurements (Figure S2 in Supporting Information shows the fitting results for additional outputs using this model). The fitting results provided parameters (detailed in Tables S1 and S2, Supporting Information) necessary to calculate the effective time constants (*τ*) for each configuration (see Supporting Information for the calculation method). The average effective time constants (Figure 2F) were comparable across all configurations, with the YMO_⊥,o_ configuration generally exhibiting slightly larger time constants. This suggests that the YMO_⊥,o_ configuration may have a better MC, which is advantageous for time‐series prediction in PRC applications.

A direct comparison between the YMO_⊥,s_ and YMO_//,s_ configurations reveals that the semicircle corresponding to YMO_//,s_ is noticeably larger. This suggests a higher total impedance in the parallel configuration, likely due to the presence of more insulating domain walls. This difference is further accentuated in the opposite‐side configurations: YMO_//,o_ exhibits a significantly larger semicircle compared to YMO_⊥,o_, implying stronger impedance effects from insulating domain wall arrangements encountered in the longer transport paths. Conversely, the YMO_⊥,o_ configuration likely supports more effective carrier propagation along conductive domain walls, benefiting from favorable polarization alignments and domain wall geometries, as has been previously reported for improper ferroelectrics such as YMnO_3_.^[^ ^22^, ^23^ ^]^

Despite these variations in semicircle size and absolute impedance, the extracted effective time constants (Figure 2F) were broadly comparable across all configurations. This suggests that the timescales of dielectric relaxation and charge carrier dynamics are relatively stable and governed by intrinsic properties rather than by macroscopic geometric factors. This apparent invariance in time constants may arise because charge transport preferentially occurs along energetically favorable conductive domain walls rather than across insulating domains or their interfaces. As a result, the YMO_⊥,o_ configuration, which appears to support more efficient in‐plane transport, exhibited a slightly larger average time constant, possibly due to additional capacitive effects at junctions of complex domain‐wall networks or extended relaxation paths intrinsic to the opposite‐side geometry. Overall, these results reinforce the conclusion that while domain configuration and crystal orientation modulate impedance magnitude, the key dynamical features relevant for PRC remain consistent.

Figures S3–S6 (Supporting Information) demonstrate the phase shift and high‐dimensionality properties of the YMnO_3_ devices, which are essential for their application in PRC. The voltage–time (*V*–*t*) curves (Figure S3A, Supporting Information) show distinct phase shifts between the input and output signals, a crucial feature of PRC as it enables temporal processing and memory retention. Fast Fourier transform (FFT) analysis (Figure S3B, Supporting Information) revealed the presence of higher harmonics in the output signals, indicating the system's capability to transform input signals into higher‐dimensional representations—an essential factor for enhancing the computational power of the reservoir. Lissajous plots of YMO_⊥,s_ (Figure S4A, Supporting Information), used as an example, show bending and narrowing with increasing input amplitude, indicating a rise in nonlinearity and a reduction in phase shift. This increase in nonlinearity was further confirmed by power spectral density (PSD) analysis, which shows a rise in PSD with increasing input amplitude (Figure S4B, Supporting Information). Similar trends were observed in other device configurations (Figure S4C–F, Supporting Information). When the input amplitude was set to ±5 V, the Lissajous plots displayed comparable shapes across different device configurations (Figure S5A, Supporting Information), reflecting similar levels of nonlinearity and phase shifts. Consequently, similar PSD values were observed (Figure S5B, Supporting Information). Notably, YMO_⊥_ devices generally exhibited slightly higher PSD values than YMO// devices, regardless of the measurement configuration. This trend was consistent across other input amplitudes as well (Figure S5D and F, Supporting Information).

### 2.3 Thermally Stable Nonlinear I–V Characteristics

**Figure 3:**
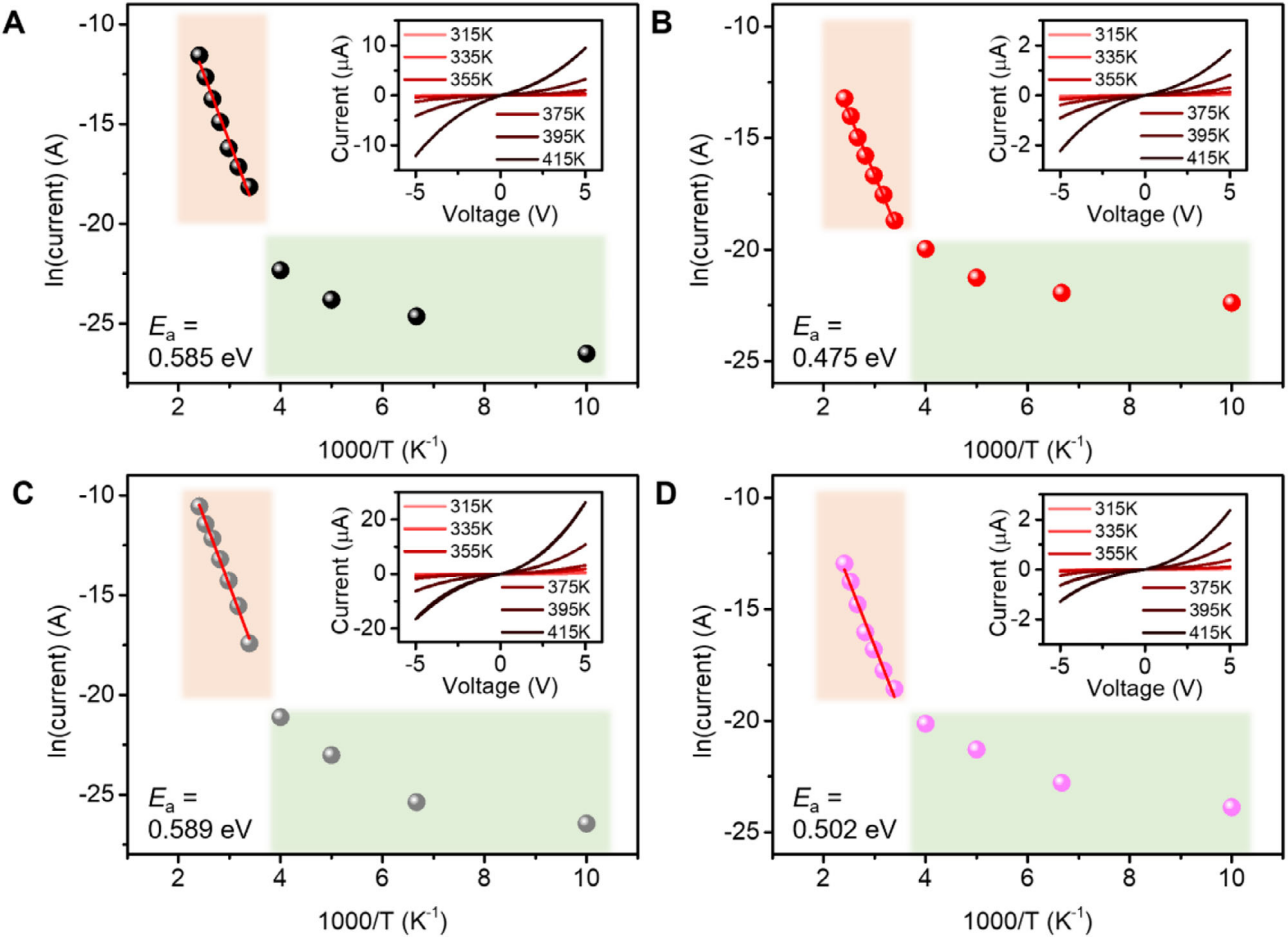
Temperature‐dependent conductivity analysis. Arrhenius plots of ln(current) versus 1/T for: A) YMO_⊥,s_ B) YMO_//,s_ C) YMO_⊥,o_, and D) YMO_//,o_. Current values were extracted from *I*–*V* plots of output 2 at +5 V. Activation energies were calculated from linear fits in high‐temperature region (T > 295 K). Each inset shows corresponding *I*–*V* plots measured at hightemperature regions.

Temperature‐dependent *I*–*V* measurements were conducted to further elucidate the charge transport mechanisms in YMnO_3_, with a focus on the material's stability and nonlinear behaviour across a wide temperature range. These measurements demonstrated strong nonlinearity above 200 K (Figure S6 (Supporting Information) and insets in **Figure** **3**), which persisted at higher temperatures, indicating robust thermally activated transport. By constructing Arrhenius plots of ln(current) versus 1/*T* (Figure 3), the activation energies for charge conduction were extracted, with values ranging from 0.475 to 0.589 eV, depending on the device configuration. As calculated by Ruff et al., charge transport through domains follows the Arrhenius law with an activation energy of 0.36 eV. However, in domain walls (less conducting), charge transport deviates from thermally activated behavior and shows variable range hopping.^[^ ^27^ ^]^ Therefore, our calculated values are reasonable for the co‐effect of domain/domain walls. Notably, devices with parallel configurations (YMO_//,s_ and YMO_//,o_), where the current flows along the crystallographic c‐axis, exhibited lower activation energies than their perpendicular counterparts (YMO_⊥,s_ and YMO_⊥,o_), where current flows across the basal ab‐plane. This anisotropy is closely tied to the orientation and type of domain walls in YMnO_3_. Ferroelectric domain walls in this material often align along the c‐axis and include tail‐to‐tail, head‐to‐head, or neutral (side‐by‐side) configurations. Among these, tail‐to‐tail domain walls are known to be the most conductive due to hole accumulation that compensates the negative bound charges, forming quasi‐one‐dimensional conduction channels.^[^ ^23^ ^]^ In the parallel configurations, the current path is more likely to align with and travel along such conductive tail‐to‐tail domain walls, where carriers experience reduced potential barriers and less thermally activated hopping. This results in lower effective activation energies. Conversely, in perpendicular configurations, the current must traverse across domains or intersect less conductive domain wall types—such as head‐to‐head or neutral walls—where conduction is impeded by depleted carrier densities or absence of bound charges. The discontinuity and increased resistivity of these paths necessitate more thermally activated transport, leading to higher activation energies.

The thermal stability of YMnO_3_ single crystals is crucial for integration into CMOS‐compatible chips, which often undergo high‐temperature fabrication processes. The material's ability to maintain its nonlinear behaviour under such conditions indicates its suitability for energy‐efficient PRC applications, particularly in environments requiring high thermal resilience. This stability makes YMnO_3_ an attractive candidate for integrated circuit technologies, where consistent performance across a wide temperature range is essential.

The strong nonlinearity, SCLC characteristics, and thermally activated behaviour with moderate activation energies collectively highlight the rich, tuneable transport dynamics in YMnO_3._ These properties can facilitate high‐dimensional, nonlinear mapping of inputs, making YMnO_3_ a promising material for PRC applications.

### 2.4 Power Efficiency

To demonstrate the power efficiency of the fabricated YMnO_3_ devices, we estimated their power consumption based on the measured *I*–*V* characteristics shown in Figure 2A–D. The results indicate that an average current of 23.6 nA flowed under an applied voltage of 5 V, resulting in a total power consumption of approximately 1.77 µW for 15 output channels. This is significantly lower compared to other reported PRC systems, such as a dynamic memristor‐based RC system (approximately 22 µW),^[^ ^9^ ^]^ an Ag‐Ag_2_S NP memristor‐based RC system (approximately 13.81 µW),^[^ ^12^ ^]^ an Ag_2_Se device (264 µW),^[^ ^13^ ^]^ a photonic RC system (23 mW),^[^ ^28^ ^]^ and a memristive nanowire networks‐based PRC system (75 mW).^[^ ^29^ ^]^

In addition to total power consumption, power consumption per computational node is crucial for evaluating the power efficiency of a device. In this domain structure‐based PRC system, each ferroelectric domain functions as an equivalent node, similar to a virtual node in conventional reservoir computing systems. From Figure S1A (Supporting Information), the domain density on the YMO_⊥_ surface was determined to be 10.88/µm^2^. Given the area of the domain network between the electrodes, the total number of domains was estimated to be approximately 9.24×10^4^. Therefore, the power consumption per single domain in the YMO_⊥,s_ device was calculated to be 0.02 nW. This is notably lower than that of other PRC systems, such as an Ag_2_Se device (0.07 nW/junction),^[^ ^13^ ^]^ a 3D‐printed composite of carbon black and polylactic acid (7 mW/neuron),^[^ ^30^ ^]^ and in an example represented by a CMOS analogue circuit (1.6–1.9 nW/synapse).^[^ ^31^ ^]^ These findings indicate that the YMnO_3_ devices fabricated in this study exhibit superior power efficiency. Moreover, the power consumption could potentially be further reduced in practical implementations by optimising the operation time.

### 2.5 Performance Evaluation in Complex Computational Tasks

#### 2.5.1 Waveform Generation

The WG task is a fundamental method to evaluate the material's performance in PRC.^[^ ^10^, ^12^, ^13^, ^14^ ^]^ In this task, an 11‐Hz sinusoidal waveform with amplitudes of ±5 V, ±10 V, or ±15 V is applied as the input signal to the material‐based reservoir. The reservoir processes the input through its intrinsic nonlinear and memory‐rich response. The resulting 15 output signals are then used to train a linear readout function by ridge regression that maps them to a desired target waveform. The targets are waveforms with varied shapes or frequencies, including cosine, sawtooth, square, triangle, sin(2ω), and sin(3ω), which test the material's ability to perform nonlinear transformation and frequency multiplication. For WG, the accuracy was calculated as follows:

(1) $$Accuracy=1-\frac{\sum_{t}{\left(y_{target\left(t\right)}-y_{output\left(t\right)}\right)}^{2}}{\sum_{t}{\left(y_{target\left(t\right)}-{\bar{y}}_{target}\right)}^{2}}$$

where *y*_output_(*t*), *y*_target_(*t*), and $\bar{y}$_target_ are the output, target, and mean of the target, respectively.

Figure S7 (Supporting Information) showcases the performance of the YMnO_3_ devices in the WG tasks. Using the YMO_⊥,s_ configuration as an example, the devices demonstrated high prediction accuracy across all WG tasks (Figure S7A, Supporting Information). An increase in input amplitude significantly improved the prediction accuracy across all device configurations (Figure S7B–E, Supporting Information). This improvement is attributed to the increase in nonlinearity and power spectral density (PSD) with higher input amplitudes, despite a decrease in phase shifts. The prediction accuracy across different device configurations was comparable at the same input amplitude, due to similar levels of nonlinearity, phase shift, and higher harmonic generation. These results underscore the critical importance of nonlinearity and harmonic generation in achieving high performance in WG tasks. The comparable prediction accuracies across different configurations at the same input amplitude indicate that YMnO_3_ devices possess robust PRC capabilities, driven by their inherent nonlinear characteristics and efficient harmonic generation.

#### 2.5.2 NARMA2 and MC

The NARMA2 task is a widely used benchmark for evaluating time‐series prediction capabilities. In this task, the target signal is constructed using the following equation:

(2) $$\begin{array}{c} y_{target}\;\left(t\right)=0.4y_{target}\left(t-1\right)+0.4y_{target}\left(t-1\right)y_{target}\left(t-2\right)\\ +0.6x^{3}\left(t-1\right)+0.1 \end{array}$$

where *y*_target_(*t*) and *x*(*t*) are the output and input at time frame *t*, respectively. The input signal *x*(t) was normalized to the range of [–1,1] from a sequence of random input pulses in the range of 0 to the input amplitude.

The performance of the NARMA2 task was evaluated by calculating the normalized mean squared error (NMSE) between the output *y*_output_ and target *y*_target_ using the following equation.

(3) $$NMSE=\frac{\sum_{t}{\left(y_{target\left(t\right)}-y_{output\left(t\right)}\right)}^{2}}{\sum_{t}{\left(y_{target\left(t\right)}-{\bar{y}}_{target}\right)}^{2}}\;$$

All NMSE values were below 0.15 across the device configurations at different input amplitudes (Figure S8A, Supporting Information). Notably, the YMO_⊥,o_ configuration showed the lowest NMSE (0.065) for the NARMA2 task, indicating an excellent fit to the target (Figure S8B, Supporting Information). This result demonstrates YMnO_3_’s strong performance in predicting dynamic time series. As the input amplitude increased, the NMSE values decreased, showing improved prediction accuracy. The NMSE values were comparable across device configurations. An optimised sampling time was determined to be 1 ms (Figure S9, Supporting Information), which was used in subsequent measurements.

MC tasks were conducted to evaluate YMnO_3_’s short‐term memory capabilities.^[^ ^32^ ^]^ In the MC task, the same input signal used for the NARMA2 task—a random sequence between 0 and 1—was applied to the reservoir. Correlation (Cor^2^) between the input and generated output at each *k* time step was calculated by the following equation:

(4) $$Cor^{2}\;\left(k\right)=\frac{cov^{2}\left(y_{target}\left(k\right),y_{output}\left(k\right)\right)}{var\left(y_{target}\left(k\right)\right)var\left(y_{output}\left(k\right)\right)}$$

where *y*_target_(*k*) is the input signal delayed by *k* time steps, and *y*_output_(*k*) is the trained output obtained by applying a linear readout to the current reservoir states. The readout weights are optimized using ridge regression to best match *y*_output_(*k*) to the target. MC is then calculated by summing all the Cor^2^ values.

When the input amplitude was 5 V, the MC values of the opposite‐side measurement configurations were higher than those of the same side configurations (Figure S8C and D, Supporting Information), mainly due to the higher Cor^2^ values at the 3rd and 4th time steps. These results corresponded to the slightly higher effective time constants of the YMnO_3_ crystallites measured from the opposite sides. As the input amplitude increased, the MC values decreased significantly for the same side measurement configurations and increased slightly for the opposite‐side configurations (Figure S8D, Supporting Information), resulting in comparable MC values fluctuating between 4.5 and 5. The MC values were comparable to previously reported ones.^[^ ^12^, ^33^ ^]^ These results underscore the effectiveness of YMnO_3_ single crystals in handling complex time‐series predictions and retaining information over time. The strong performance in both the NARMA2 and MC tasks highlights YMnO_3_’s potential as a robust material for PRC applications.

#### 2.5.3 Voice Recognition

Voice recognition tasks present significant challenges due to the high dimensionality of voice signals. Traditionally, machine learning and deep learning methods have dominated this field,^[^ ^34^, ^35^ ^]^ but they are known for their high power consumption. While some PRC (RC) systems have achieved high accuracy, they often require numerous devices and complex preprocessing steps,^[^ ^36^, ^37^ ^]^ which limits their efficiency. Recently, a random network of sulfonated polyaniline (PANI) demonstrated improved power efficiency as an in‐materio physical reservoir for voice recognition.^[^ ^10^ ^]^ However, the demand for stable and high‐performance RC systems persists.

**Figure 4:**
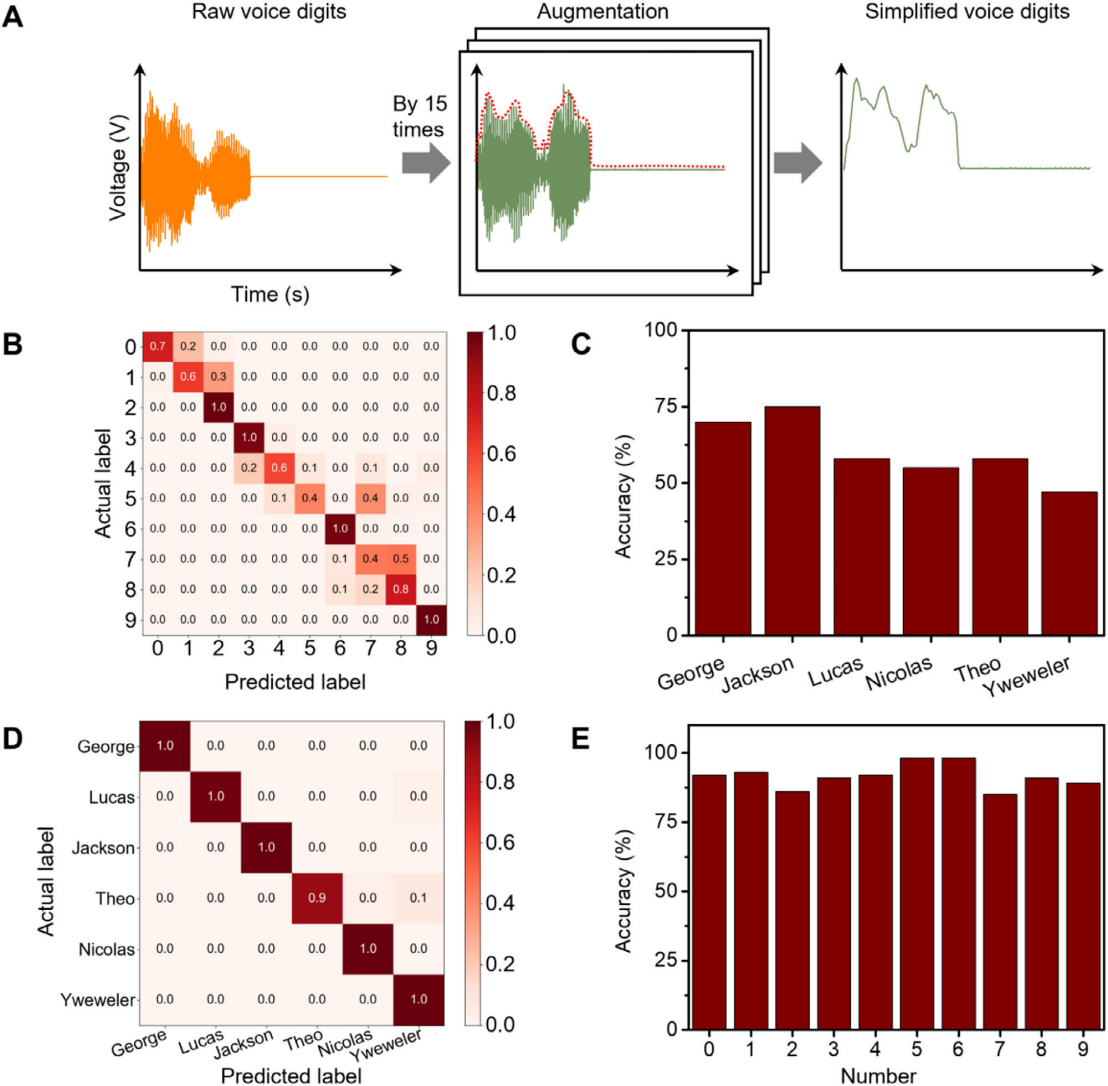
Voice recognition task performance. A) Voice pretreatment procedure. Raw voice digits were augmented by sulfonated polyaniline network as reported.^[^ ^10^ ^]^ To extract envelope of augmented voice digit signals (indicated by red dotted line), signals were rectified, down‐sampled, and normalized (called simplified voice digits). B) Recognition performance of YMO_⊥_ for 10 digits spoken by Jackson. C) Recognition accuracy of YMO_⊥_ for ten digits spoken by six speakers. D) Recognition performance of YMO_⊥_ for digit “5” spoken by six persons. E) Recognition accuracy of YMO_⊥_ for all ten digits spoken by six persons. Note: All tests were conducted with 15 V input voltage amplitude.

**Figure** **4** illustrates the application of YMnO_3_ devices in voice recognition tasks. Unlike conventional methods such as Mel frequency cepstral coefficients (MFCC)^[^ ^38^ ^]^ or cochleagram,^[^ ^10^ ^]^ this study employed a simplified preprocessing approach for raw voice digits. As shown in Figure 4A and Figure S10 (Supporting Information), the raw data were augmented 15‐fold to increase the database size. The augmented data were then rectified to the positive side and down‐sampled from 2000 to 100 points. Finally, the envelope of the augmented data was derived to create a simplified representation of the voice digit, which was normalized and used as the input. This streamlined preprocessing approach could potentially be implemented in hardware, significantly enhancing energy efficiency compared to software‐based methods. The YMO_⊥_ configuration (YMO_⊥,s_ and YMO_⊥,o_) was used as the RC system due to its excellent performance in the NARMA2 task (Table S3, Supporting Information). By comparing in terms of nonlinearity, power consumption, and task‐specific metrics (WG, MC, and NARMA2 tasks), YMO_⊥_ configuration demonstrated superiority for tasks requiring memory and nonlinear transformation. A total of 31 output signals were recorded from the YMO_⊥_‐based device: 15 signals from the same‐side electrodes and 16 from opposite‐side electrodes. These time‐series signals were labelled according to their classification target—for example, the spoken digit (0–9) or speaker. A one‐hot encoding scheme was used for the target labels: for example, the digit “3” was assigned the target vector [0, 0, 0, 1, 0, 0, 0, 0, 0, 0]. A linear classifier was trained using ridge regression to map the reservoir output features to the one‐hot targets, enabling supervised classification of digits or speakers. It demonstrated impressive recognition accuracies across different digits and speakers (Figure 4B–E), achieving up to 75% accuracy for digits and 98% accuracy for speakers. These results highlight the potential of YMnO_3_‐based PRC for practical applications in speech recognition, offering both high performance and energy efficiency. As summarized in Table S4 (Supporting Information), compared with other reported materials and technologies, multiferroic YMnO_3_ single crystals are superior in terms of high recognition accuracy, low power consumption, and good practical applicability.

### 2.6 Tuneable Computing Performance by Changing Domain Structure

Figures S11 and S12 (Supporting Information) illustrate the impact of electrical poling on the nonlinearity, phase shift, high dimensionality, and PRC performance of YMnO_3_ single crystals. The *I*–*V* characteristics in Figure S11A and B (Supporting Information) show that electrical poling results in reduced nonlinearity in the YMnO_3_ crystals, as indicated by the decreased slopes in the log‐log scale plots. This outcome is expected, as the high‐electric‐field poling process unifies the polarization directions of the domains, simplifying the domain structure and making it more linear. The simplification of the domain structure leads to a decrease in the inherent nonlinearity of the material, which is critical for effective PRC. The Lissajous plots in Figure S11C and D (Supporting Information) reveal that the phase shifts remain largely unchanged after poling. Additionally, the FFT analysis in Figure S11E and F (Supporting Information) indicates a slight decrease in PSD across various frequencies in the poled configurations, suggesting a reduced ability to generate the higher harmonics necessary for creating a high‐dimensional computational space.

In Figure S12 (Supporting Information), a comparison between poled and unpoled YMnO_3_ configurations revealed lower performance in key PRC tasks for the poled crystals. The prediction accuracy for WG (Figure S12A, Supporting Information) decreased, and the NMSE for the NARMA2 task (Figure S12B, Supporting Information) increased, indicating a decline in predictive accuracy. Despite the similar MC values for both poled and unpoled states (Figure S12C, Supporting Information), slightly lower voice recognition accuracy for the poled configurations (Figure S12D, Supporting Information) suggests that the more complex domain structures in the unpoled state contribute positively to PRC performance.

This comparison between poled and unpoled YMnO_3_ crystals underscores the importance of complex domain structures in enhancing PRC capabilities. The reduction in performance following the simplification of the domain structure highlights the critical role that these intricate domain arrangements play in achieving high computational efficiency. Moreover, these findings suggest that PRC performance can be optimised by carefully tuning the domain structure, potentially leading to even greater improvements, along with the added benefits of rewritability or programmability in computational tasks.

## 3 Conclusion

This comprehensive study established YMnO_3_ single crystals as a promising platform for robust and tuneable PRC, with a particular focus on understanding the impact of measurement configuration, thermal stability, and electrical poling on performance. Across multiple device configurations (YMO_⊥,s_, YMO_//,s_, YMO_⊥,o_, and YMO_//,o_), the unpoled YMnO_3_ crystals demonstrated robust performance in key PRC tasks, including WG, NARMA2, and MC. The unique combination of pronounced nonlinear *I*–*V* characteristics, phase shifts, and higher harmonic generation, presumably associated with the distinctive domain structure of this system, contributed significantly to the material's PRC capabilities. The thermal stability of YMnO_3_ was confirmed, and the material maintained its strong nonlinear behaviour over a wide temperature range, making it suitable for integration into CMOS‐compatible devices that often require high‐temperature fabrication processes. The power consumption of YMnO_3_ was estimated to be 1.77 µW in total and 0.02 nW per domain, outperforming many PRC systems in terms of energy efficiency. The influence of electrical poling on the performance of YMnO_3_ crystals was thoroughly evaluated, revealing that poling reduced nonlinearity due to the unification of domain polarization directions. This simplification of the domain structure led to a decrease in performance in key tasks, as evidenced by lower prediction accuracy in WG and higher NMSE in NARMA2 tasks. Despite the similar MC of the poled and unpoled samples, the poled crystals exhibited a diminished ability to generate higher harmonics, which is crucial for maintaining high‐dimensional computational spaces.

In practical applications, particularly in voice recognition tasks, the YMnO_3_‐based PRC system achieved impressive results, with recognition accuracies of up to 75% for digits and 98% for speakers. These findings not only established YMnO_3_ as a viable material for PRC but also opened new avenues for energy‐fficient, high‐performance computing in various domains.

Future research could focus on optimising device configurations, exploring the impact of domain structures on performance, and investigating multi‐reservoir systems for more complex tasks. As the pursuit of computational efficiency and capability advances, material‐based approaches, such as the one presented here, will play an increasingly crucial role in shaping the future of information processing.

## 4 Experimental Section

### Preparation and Characterization of YMnO_3_ Single Crystallites

The experimental study began with the preparation of high‐quality YMnO_3_ single crystals using the floating‐zone method.^[^ ^39^ ^]^ The single crystals were cut in two directions to obtain two types of crystallites: YMO_⊥_ and YMO_//_, with surfaces perpendicular and parallel to the crystallographic *c*‐axis, respectively. Each crystallite had a thickness of approximately 1.5 mm. For poling the YMO_⊥_ crystallites (with approximately 800 µm thickness), Cu electrodes were placed on the c‐plane surfaces of the plate‐like crystals, and large electric fields of approximately 75 kV/cm were applied to the symmetric electrodes. Specimens for transmission electron microscopy (TEM) were prepared from YMnO_3_ single crystal by Ar ion milling. TEM observations were carried out with a JEOL‐F200 operating at 200 kV at room temperature.

### Fabrication of YMnO_3_‐Based Reservoir Device

The YMnO_3_ crystallite (YMO_⊥_ or YMO_//_) was securely fixed into a central hole of a printed circuit board (PCB) using a heat‐resistant adhesive paste. Then, 16 gold (Au) electrodes were deposited on the front and back surfaces of the crystallite via thermal evaporation at a rate of 1.5 Å s^−1^ to a thickness of 100 nm. The Au electrodes were then bonded to the conductive pads on the PCB using Au wires and silver paste to enable electrical probing.

### Electrical Measurements

A comprehensive suite of electrical characterization techniques was employed to thoroughly assess the PRC capabilities of the YMnO_3_ crystals across different device configurations. *I*–*V* measurements were performed to probe the nonlinear transport characteristics, a key requirement for PRC. Both room‐ and high‐temperature *I*–*V* measurements were performed using a Keithley 2636 source meter controlled by a custom LabVIEW programme. The temperature was regulated with a hot plate. For low‐temperature *I*–*V* measurements, a probe system (Pascal Co., Ltd.) combined with an Agilent 4156 B semiconductor parameter analyser was utilized.

Electrochemical impedance spectra were measured using a Zurich MFIA impedance analyser, with the results represented as Cole‐Cole plots. In these plots, the imaginary impedance (Z″) was plotted against the real impedance (Z′), derived from the absolute impedance and phase angle measurements during a frequency scan ranging from 1 Hz to 5 MHz. The DC bias voltage was set at +5 V, and the AC amplitude was 0.3 V. Cole‐Cole plots were fitted using the free EIS analyser software, eissa1.

For time‐domain voltage–current (*V*–*t*) measurements, a multifunction data acquisition (DAQ) system (National Instruments PXIe‐6363 connected to SCB‐68A) was utilized, controlled via LabVIEW software for input signal generation and output data recording. This method was consistent with previously reported techniques.^[^ ^12^ ^]^ The measurements were conducted at a sampling rate of 1000 points/s. The inputs and outputs were measured simultaneously. The *V*–*t* plots generated from an 11‐Hz sinusoidal wave input were analysed to investigate phase shifts, higher harmonics, and WG accuracy. *V*–*t* plots generated from white‐noise input were analysed for the NARMA2 and MC tasks.

A free‐spoke‐digit‐data dataset^[^ ^40^ ^]^ containing voice recordings of the numbers zero to nine, pronounced 47 times each by six speakers (George, Lucas, Jackson, Theo, Nicolas, and Yweweler), was used for voice recognition. This dataset was augmented 15 times using a sulfonated polyaniline network,^[^ ^10^ ^]^ followed by rectification and downsampling with Python. The simplified voice signals were then normalized and applied to the YMnO_3_ PRC system as time‐series bias voltages using LabVIEW software and an amplifier (Thurlby Thandar Instruments, WA301 Wide Band Amplifier, 30V pk‐pk). The output signals from the device were recorded, and all signals were labeled as real numbers for classification. A one‐hot vector was used as the target to optimise the voice digit classification. Ridge regression was applied for WG, MC, NARMA2, and voice recognition tasks using Python software. Unless otherwise specified, all measurements were performed at a sampling rate of 1000 points/s.

### YMnO_3_‐Based Physical Reservoir Computing

The physical reservoir architecture was based on a multiferroic YMnO_3_ single crystal equipped with 16 electrodes arranged in a spider‐like configuration (Figure S1D, Supporting Information) on top and bottom sides. As shown in Figure 1A, an external input signal *x*(*t*) (e.g., a voltage waveform) was applied to one electrode, and the device's nonlinear and time‐dependent response was collected from the remaining 15 electrodes on the same side and 16 electrodes on the opposite side as time‐varying output signals *r_i_* (*t*). These signals form the high‐dimensional reservoir states at each time step *t*. To map the measured reservoir states *r_i_* (*t*) to the target output, y_target_(*t*), a linear readout layer was trained using ridge regression. The predicted output y_output_(*t*), was computed as a linear combination of *r_i_* (*t*) weighted by a readout vector *W*_out_, where *W*_out_ = [*W*_1_, *W*_2_, …, *W_i_*]^T^. For WG, NARMA2, and MC tasks, *i* = 15. For voice recognition task, *i* = 31.

## Conflict of Interest

The authors declare no conflict of interest.

## Supporting information



Supporting Information

## Data Availability

The data that support the findings of this study are available from the corresponding author upon reasonable request.
