# Supplementary material for: Intrinsic Disordered Network in Multiferroic YMnO3 Single Crystals for In‐Materio Physical Reservoir Computing Through Tuneable Domain‐Wall Structure
Source: Small. 2025 Sep 12;21(48):e06397. doi: 10.1002/smll.202506397 (PMC12674103; doi:10.1002/smll.202506397)
Supplement: Supplementary file 1 — Supporting Information [file SMLL-21-e06397-s001.docx]

Supporting Information

Intrinsic Disordered Network in Multiferroic YMnO₃ Single Crystals for In-Materio Physical Reservoir Computing through Tuneable Domain-wall Structure

Muzhen Xu^1^, Kyoka Furuta^2^, Ahmet Karacali^3^, Yuki Umezaki^2^, Alif Syafiq Kamarol Zaman^3^, Yuki Usami^1,3^, Hirofumi Tanaka*^1,3^, and Yoichi Horibe*^1,2^

1. Research Center for Neuromorphic AI Hardware, Kyushu Institute of Technology, 2-4 Hibikino, Wakamatsu, Kitakyushu, 808-0196 Japan

2. Department of Materials Science and Engineering, Kyushu Institute of Technology, 1-1 Sensui, Tobata, Kitakyushu 804-8550, Japan

3. Department of Human Intelligence Systems, Kyushu Institute of Technology, 2-4 Hibikino, Wakamatsu, Kitakyushu, 808-0196 Japan

E-mail: horibe@post.matsc.kyutech.ac.jp; tanaka@brain.kyutech.ac.jp

**Method to estimate effective time constants**

The impedance of CPE*_i_* (Z_CPE,_*_i_*) (*i* = 1, 2, 3) is described as^1^

$$Z_{CPE,i}=\frac{1}{P_{i}{(jw)}^{n_{i}}}$$

, where *P_i_* is a parameter containing capacitance information, *w* means angular frequency, *j* is the imaginary unit, and *n_i_* is a constant ranging from 0 to 1. The exponent *n_i_* describes the deviation from idea capacitor performance. *n_i_* = 1 indicates CPE*_i_* behaves as an ideal capacitor. And *n_i_* = 0 indicates CPE*_i_* behaves as a resistor.

For each R*_i_*_+1_CPE*_i_* (*i* = 1, 2, 3) circuit, the effective time constant *τ*_i_ was estimated as

$\tau_{i}\approx R_{i+1}P_{i}$ (1),

when *n* ≥ 0.7, which means that the CPE element was regarded as a capacitor. *R_i_*_+1_ means the resistance of resistor R*_i_*_+1_. For 0 < *n* <0.7, the CPE element was regarded as a resistor, and *τ_i_* = 0 s. The total effective time constant was calculated as a summarisation of all *τ*.

[1] A. C. Lazanas and M. I. Prodromidis, *ACS Measurement Science Au*, 2023, **3**, 162-193.


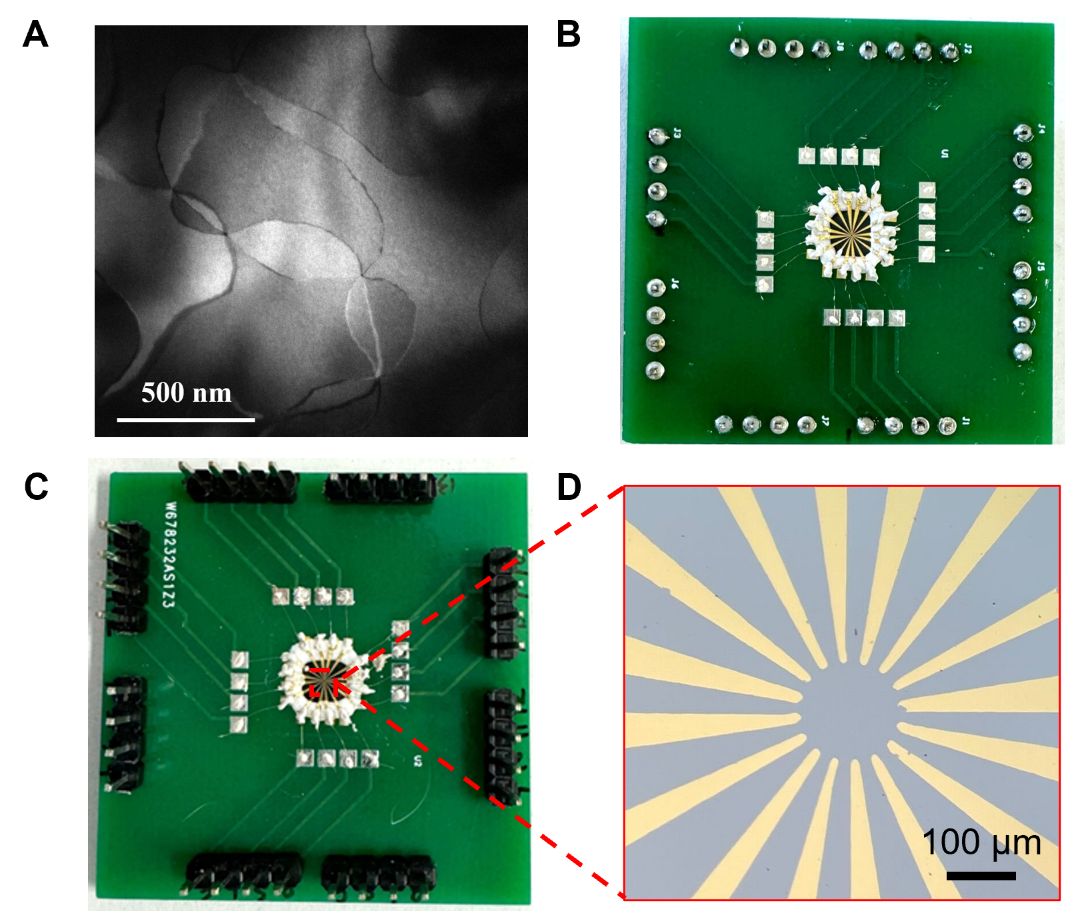


**Figure S1.** YMnO_3_ single crystal-based in-materio PRC device. (A) Transmission electron microscopy (TEM) image of YMO_⊥_ single crystal, showing cloverleaf-like domain structure. (B-D) Photographs of in-materio PRC device: (B) front view of device, (C) back view of device, and (D) microscope image showing YMnO_3_ single crystallite embedded in centre of PCB board with 16 Au electrodes deposited on its surface.


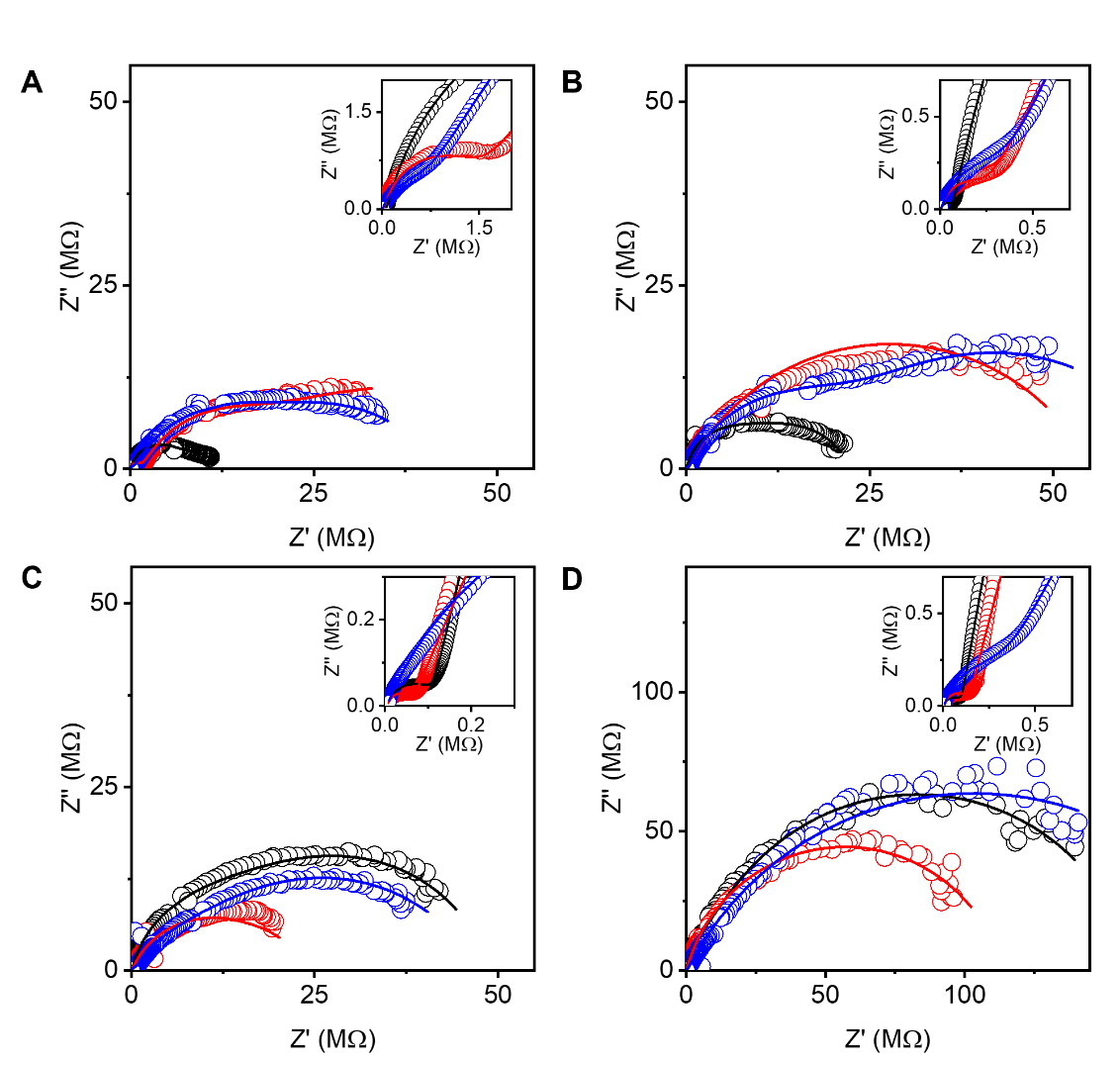


**Figure S2.** Cole-Cole plots of YMO_⊥,s_ (A), YMO_//,s_ (B), YMO_⊥,o_ (C), and YMO_//,o_ (D), respectively. Each inset shows magnified high-frequency region correspondingly. Dots: data points. Line: fitting results. Black: output 1. Red: output 4. Blue: output 8.

**Table S1.** Fitting parameter values of each element in equivalent circuit.

| Device configuration | YMO_⊥,s_ | | | YMO_//,s_ | | | YMO_⊥,o_ | | | YMO_//,o_ | | |
| --- | --- | --- | --- | --- | --- | --- | --- | --- | --- | --- | --- | --- |
| Output no. | 1 | 4 | 8 | 1 | 4 | 8 | 1 | 4 | 8 | 1 | 4 | 8 |
| R_1_ (kΩ) | 5.07 | 1.00 | 4.72 | 5.56 | 1.02 | 6.33 | 5.94 | 6.31 | 4.35 | 6.79 | 5.47 | 1.31 |
| R_2_ (MΩ) | 0.56 | 1.09 | 0.60 | 0.59 | 0.20 | 0.30 | 0.10 | 0.53 | 0.61 | 0.89 | 0.13 | 0.24 |
| R_3_ (MΩ) | 5.69 | 10.00 | 20.8 | 7.89 | 55.1 | 45.2 | 35.2 | 3.19 | 7.29 | 5.92 | 114 | 207 |
| R_4_ (MΩ) | 6.53 | 54.50 | 19.8 | 14.4 | 50.1 | 21.7 | 13.5 | 19.9 | 39.4 | 155 | 52.1 | 100 |
| P_1_ (pΩ^-1^ s^n1^) | 19.8 | 6.95 | 112 | 20.8 | 8.92 | 25.4 | 24.5 | 12.0 | 143 | 16.1 | 9.21 | 28.2 |
| n_1_ | 0.93 | 1.00 | 0.80 | 0.92 | 0.96 | 0.90 | 0.89 | 0.95 | 0.79 | 0.92 | 0.94 | 0.90 |
| P_2_ (nΩ^-1^ s^n2^) | 0.46 | 1.00 | 5.96 | 0.39 | 1.06 | 3.35 | 1.73 | 38.0 | 1.24 | 0.46 | 0.45 | 0.77 |
| n_2_ | 0.92 | 0.85 | 0.71 | 0.93 | 0.70 | 0.73 | 0.83 | 1.00 | 0.73 | 0.99 | 0.84 | 0.70 |
| P_3_ (nΩ^-1^ s^n3^) | 14.1 | 10.6 | 0.72 | 3.16 | 1.75e^5^ | 0.38 | 0.60 | 2.36 | 2.19 | 0.42 | 4.46e^5^ | 2.64e^5^ |
| n_3_ | 0.65 | 0.48 | 0.73 | 0.77 | 4.4e^-10^ | 0.78 | 0.88 | 0.75 | 0.69 | 0.87 | 1.0e^-13^ | 2.0e^-18^ |

**Table S2.** Effective time constants calculated based on values in Table S1.

| Device configuration | YMO_⊥,s_ | | | YMO_//,s_ | | | YMO_⊥,o_ | | | YMO_//,o_ | | |
| --- | --- | --- | --- | --- | --- | --- | --- | --- | --- | --- | --- | --- |
| Output no. | 1 | 4 | 8 | 1 | 4 | 8 | 1 | 4 | 8 | 1 | 4 | 8 |
| *τ*_1_ (μs) | 11 | 8 | 67 | 12 | 2 | 8 | 3 | 6 | 87 | 14 | 1 | 7 |
| *τ*_2_ (ms) | 3 | 10 | 124 | 3 | 58 | 151 | 61 | 121 | 9 | 3 | 51 | 159 |
| *τ*_3_ (ms) | 92 | - | 14 | 46 | - | 8 | 8 | 47 | 86 | 65 | - | - |
| *τ*_total_ (ms) | 95 | 10 | 138 | 49 | 58 | 159 | 69 | 168 | 95 | 68 | 51 | 159 |

"–" indicates that the CPE exponent *n_i_* < 0.7, and the element was regarded as a resistor with no defined capacitive time constant.


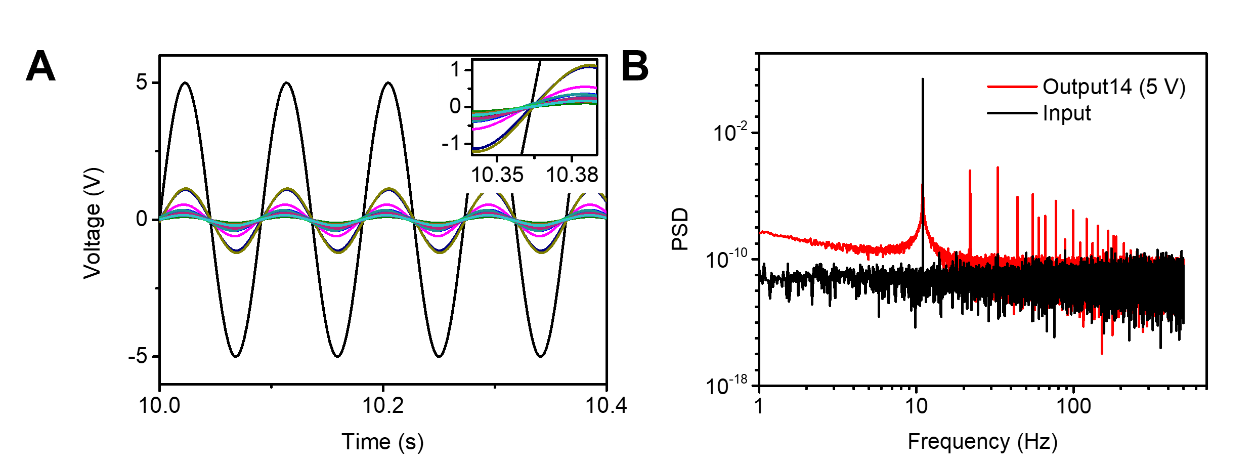


**Figure S3.** Phase shift and high dimensionality. (A) *V–t* curves for YMO_⊥,s_ configuration. Input signal: 11 Hz sinusoidal, ±5 V (black). 15 nonlinear outputs (various colours). Inset: magnified view showing phase shift between input and outputs. (B) fast Fourier transform (FFT) spectrum of *V–t* plots for YMO_⊥,s_ device configuration, showing PSD and frequency in log-log scale. Input: black line. Output 14: red line.


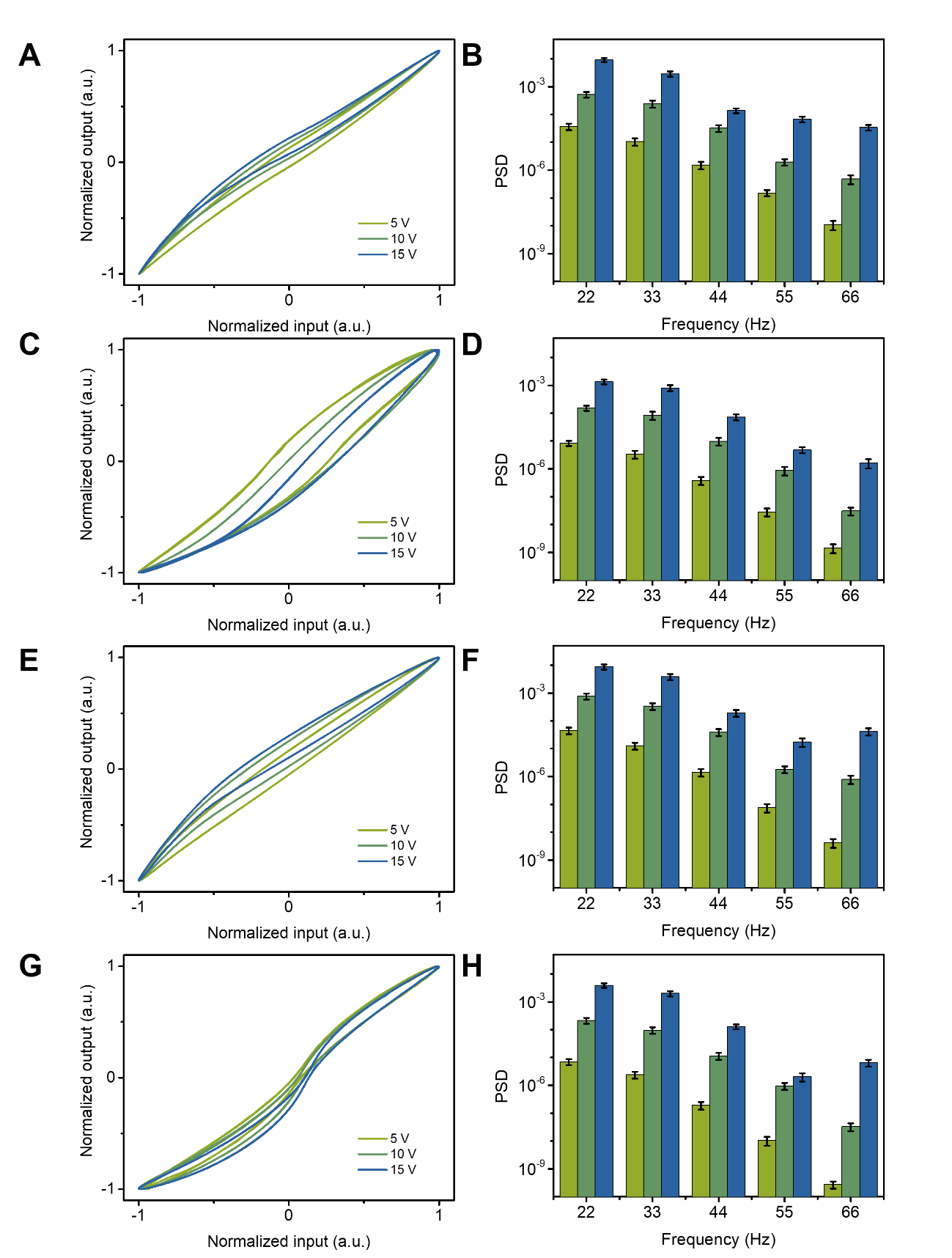


**Figure S4.** Effect of device configuration on phase shift and high dimensionality. (Left) Lissajous plots of normalised input and output 14’s voltage for YMO_⊥,s_ (A), YMO_//,s_ (C), YMO_⊥,o_ (E), and YMO_//,o_ (G), respectively. (Right) Corresponding PSD at particular frequencies obtained from FFT spectrum of *V*–*t* plots of all outputs for YMO_⊥,s_ (B), YMO_//,s_ (D), YMO_⊥,o_ (F), and YMO_//,o_ (H), respectively. Amplitudes of inputs were ±5 V (yellowish-green), ±10 V (green), and ±15 V (blue). Error bar: standard error, N = 15.


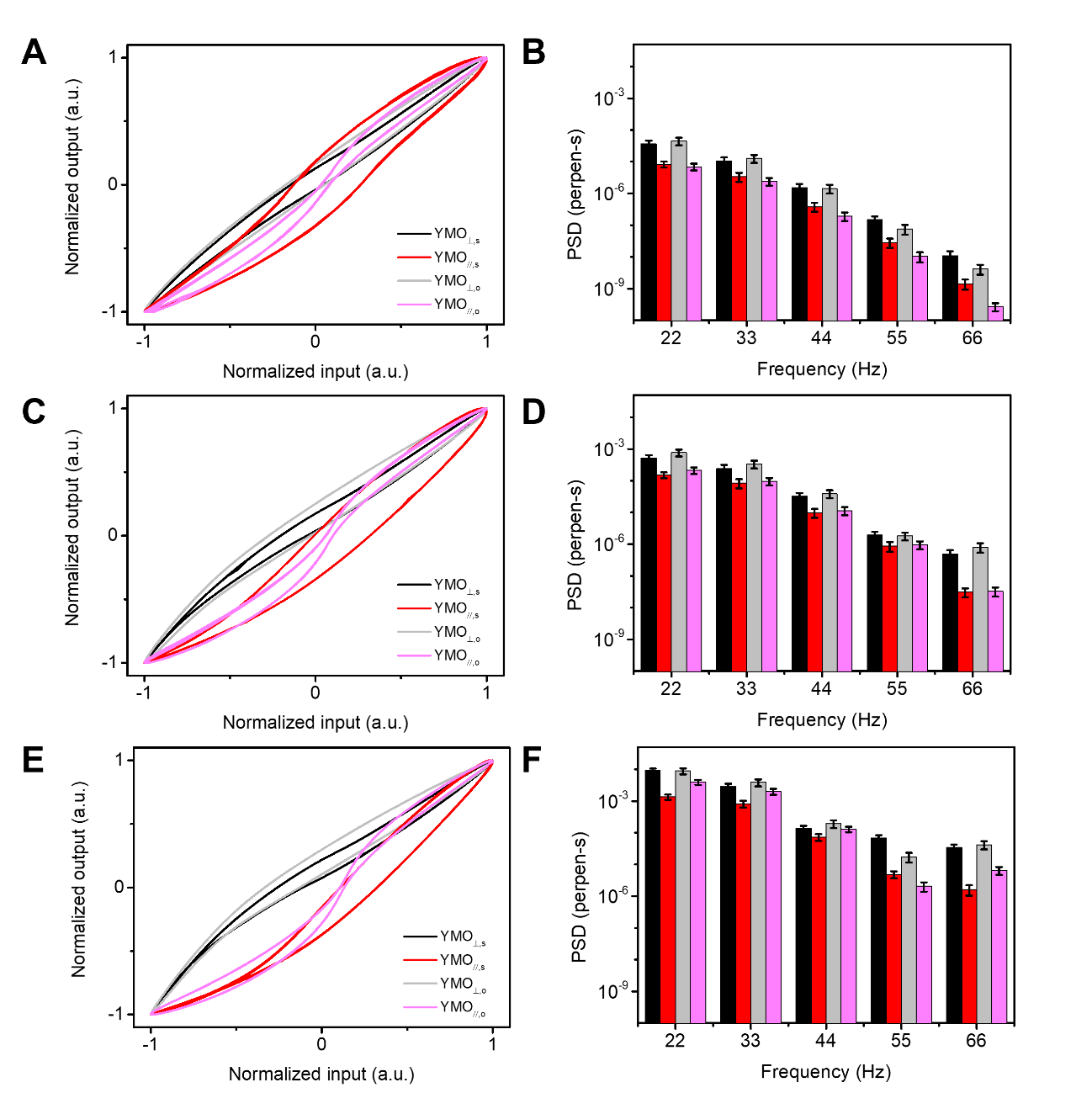


**Figure S5.** Effect of input voltage on phase shift and high dimensionality. (Left) Lissajous plots of normalised input and output 14’s voltage across all device configurations when amplitude of input was ±5 V (A), ±10 V (C), and ±15 V (E), respectively. (Right) Corresponding PSD at particular frequencies obtained from FFT spectrum of *V*–*t* plots of all outputs across all device configurations when amplitude of input was ±5 V (B), ±10 V (D), and ±15 V (F), respectively. Error bar: standard error, N = 15.


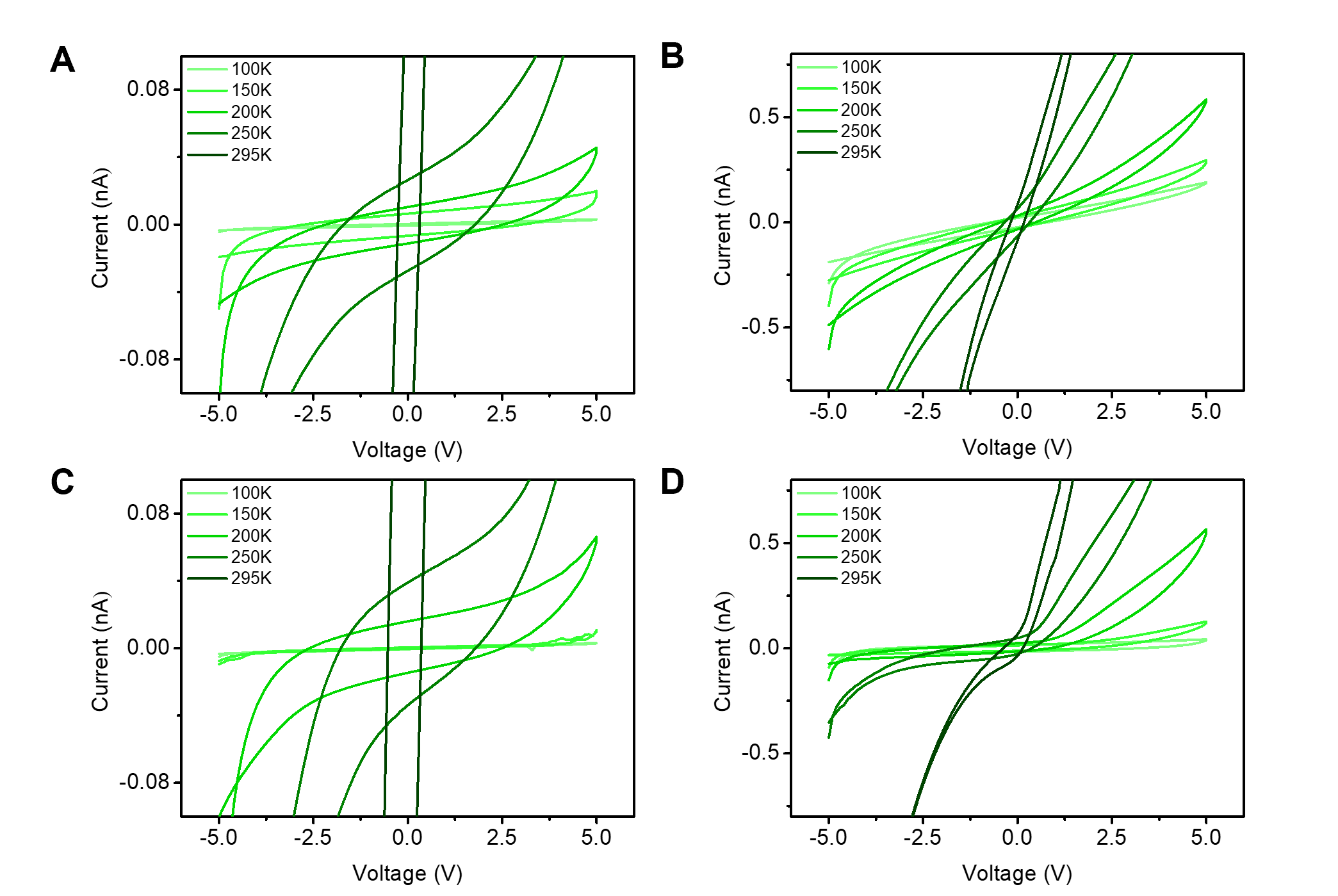


**Figure S6.** *I–V* plots measured at low-temperature region for YMO_⊥,s_ (A), YMO_//,s_ (B), YMO_⊥,o_ (C), and YMO_//,o_ (D), respectively.


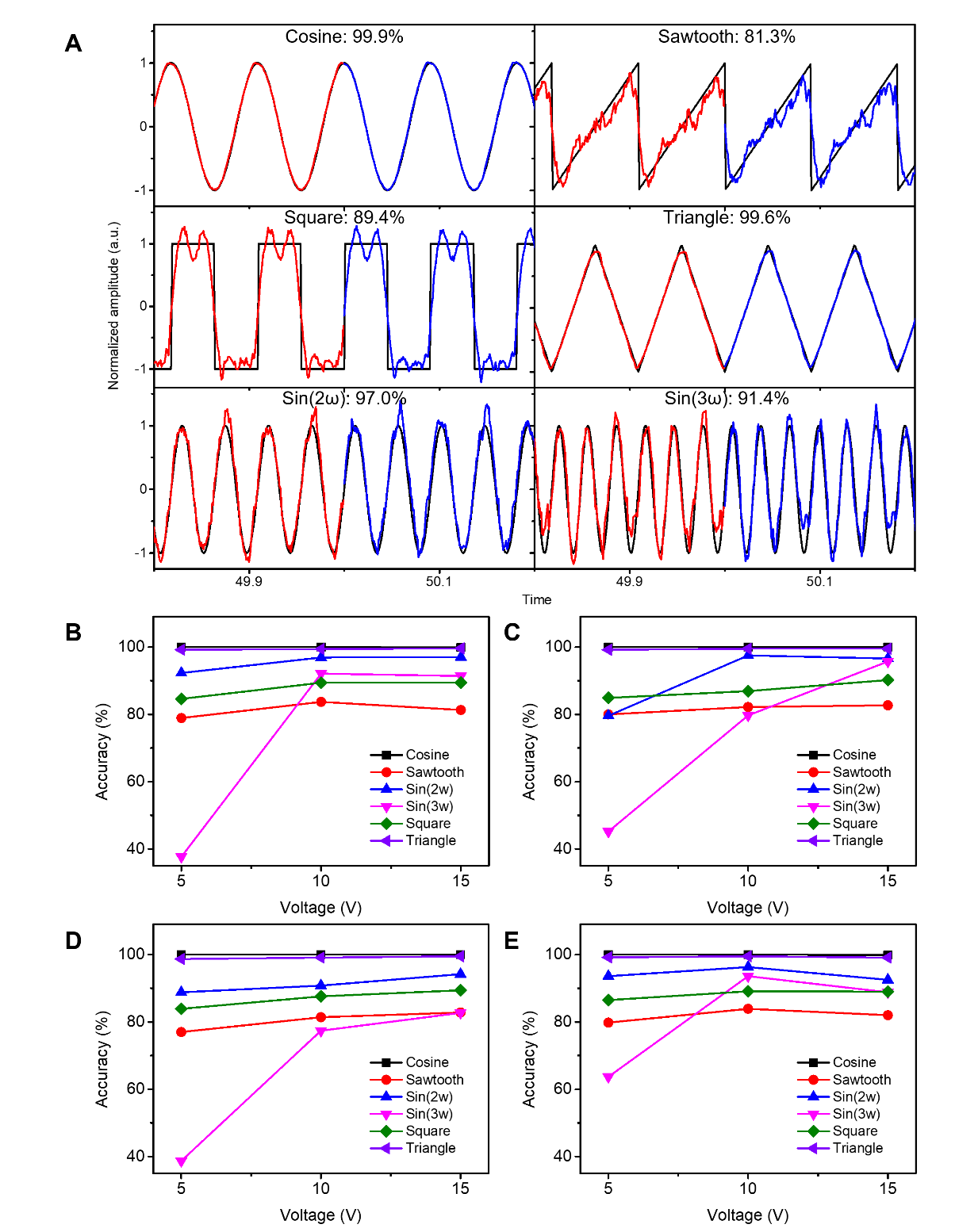


**Figure S7.** Waveform generation task performance. (A) Predicted performance (red line: training, blue line: testing) of YMO_⊥,s_ to target waveform signals (black line). Input amplitudes: ±15 V. (B-E) Prediction accuracy for all waveforms: YMO_⊥,s_ (B), YMO_//,s_ (C), YMO_⊥,o_ (D), and YMO_//,o_ (E).


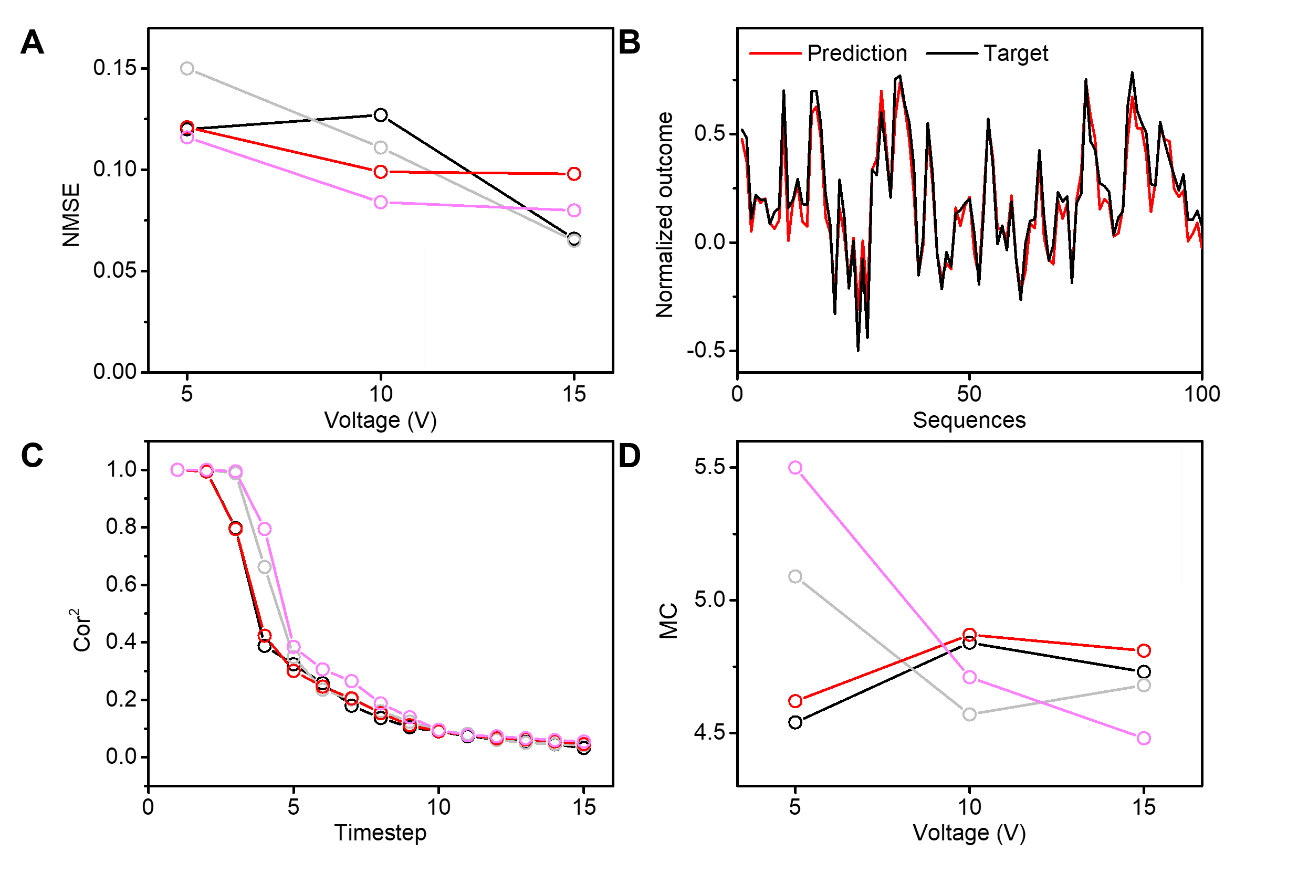


**Figure S8.** NARMA2 and MC tasks performance. (A) Normalised mean square error (NMSE) of NARMA2 task vs. amplitude of input voltage. YMO_⊥,s_ (black), YMO_//,s_ (red), YMO_⊥,o_ (light grey), and YMO_//,o_ (light magenta). (B) Predicted performance of YMO_⊥,o_ for NARMA2 task. Input amplitude: 15 V. (C) Correlation vs. timestep. Device configurations: same as in (A). (D) MC values vs. input amplitude. Device configurations: same as in (A).


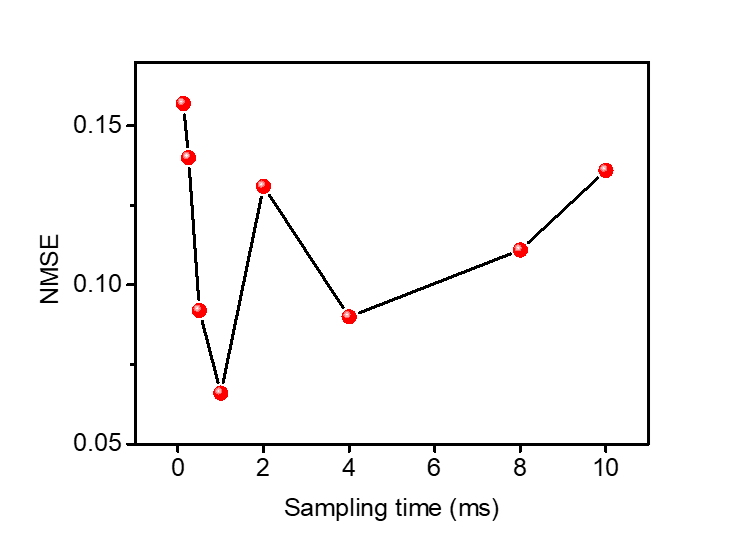


**Figure S9.** NARMA2 prediction performance of YMO_⊥,s_ for different sampling times. Amplitude of input was ±15 V.


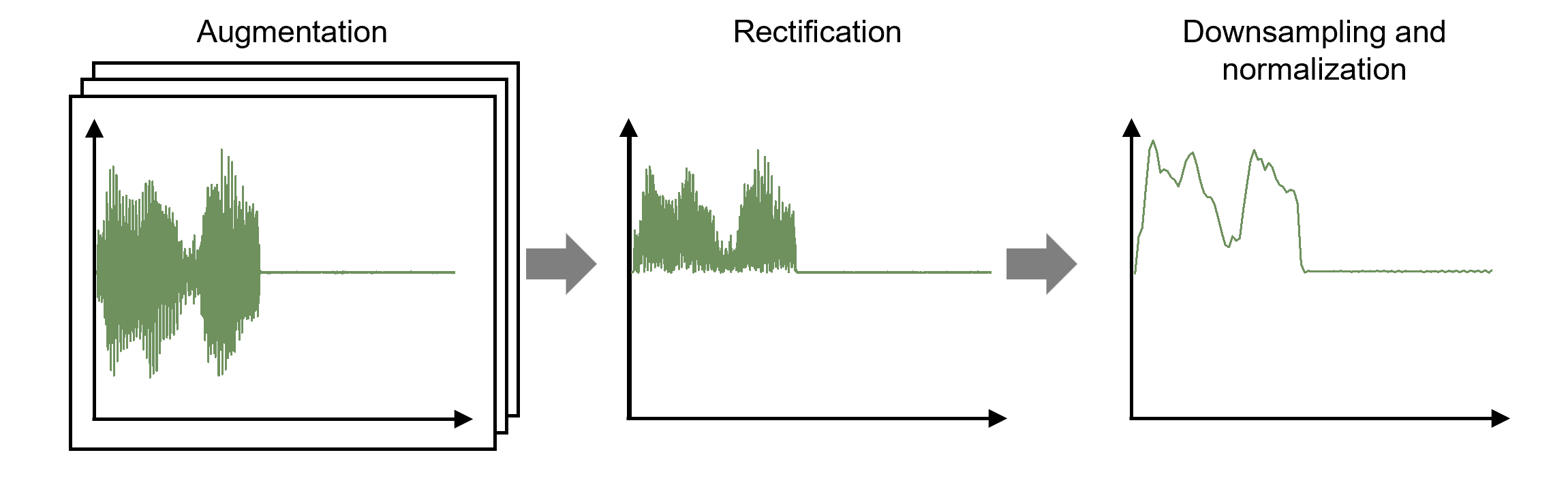


**Figure S10.** Procedure to simplify augmented voice signal in two steps. First, augmented voice signal was rectified to positive values. Second, sample points were reduced from 2000 to 100 by resampling. After this, simplified voice signal describing envelope of augmented voice signal was obtained.


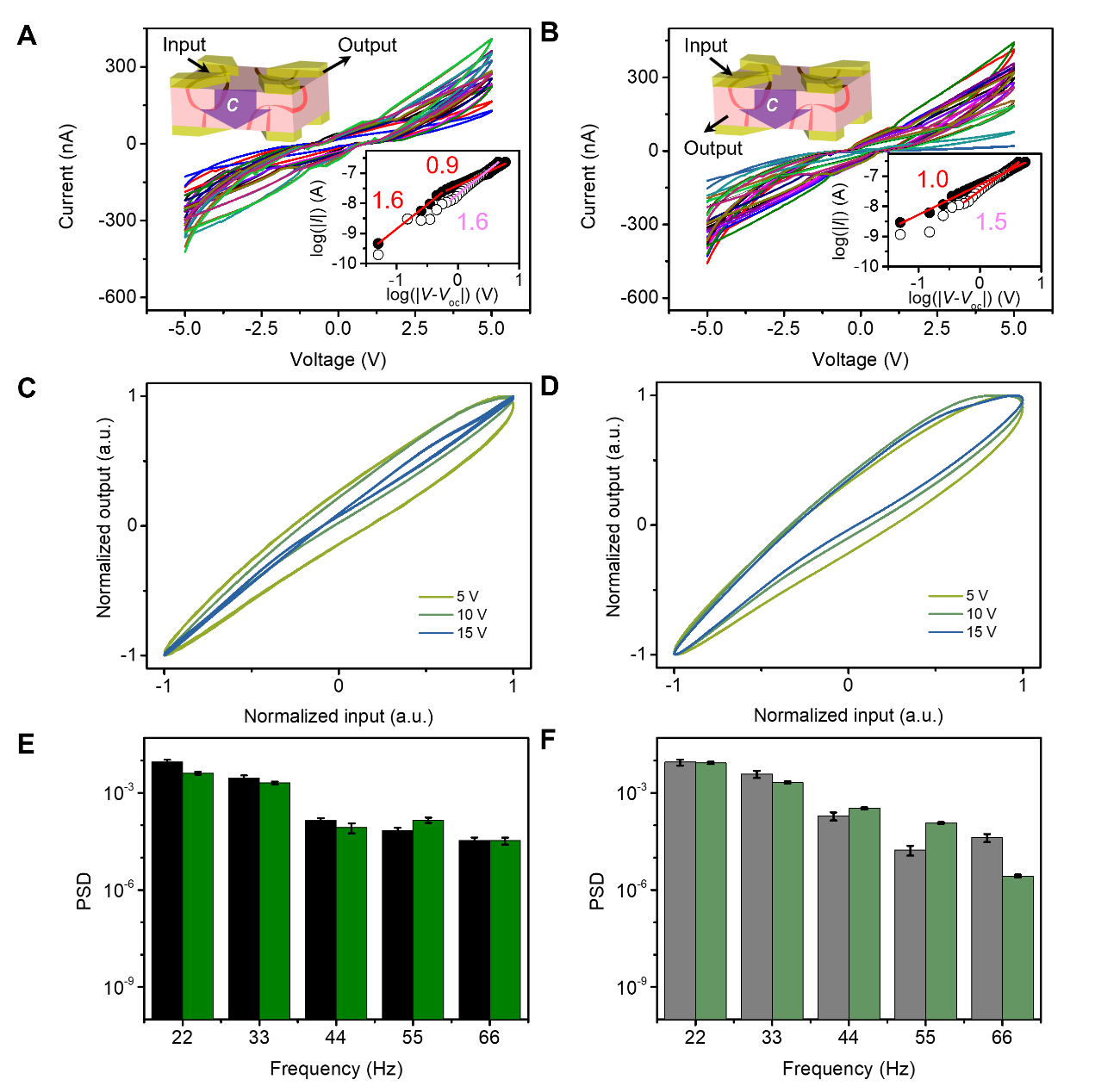


**Figure S11.** Effect of electrical poling on nonlinearity, phase shift, and high dimensionality. (A-B) 15 *I*–*V* output signals were measured for (A) YMO_⊥,s_-poled and (B) YMO_⊥,o_-poled. Each top-left inset shows corresponding schematic of device configuration. Each bottom-right inset figure shows two corresponding log-log scale *I*–*V* plots of output 1 (dot: forward scan; circle: backward scan). Each plot is linearly fitted to extract slope parameters, colour-coded, and labelled accordingly. (C-D) Lissajous plots of normalised input and output 14’s voltage for (C) YMO_⊥,s_-poled and (D) YMO_⊥,o_-poled under different amplitudes of input. (E-F) Comparison of PSD at particular frequencies obtained from FFT spectrum of *V*-*t* plots of all outputs for (E) YMO_⊥,s_ (black) and YMO_⊥,s_-poled (green) and (F) YMO_⊥,o_ (grey) YMO_⊥,o_-poled (light green). Amplitudes of inputs were ±15 V. Error bar: standard error, N = 15.


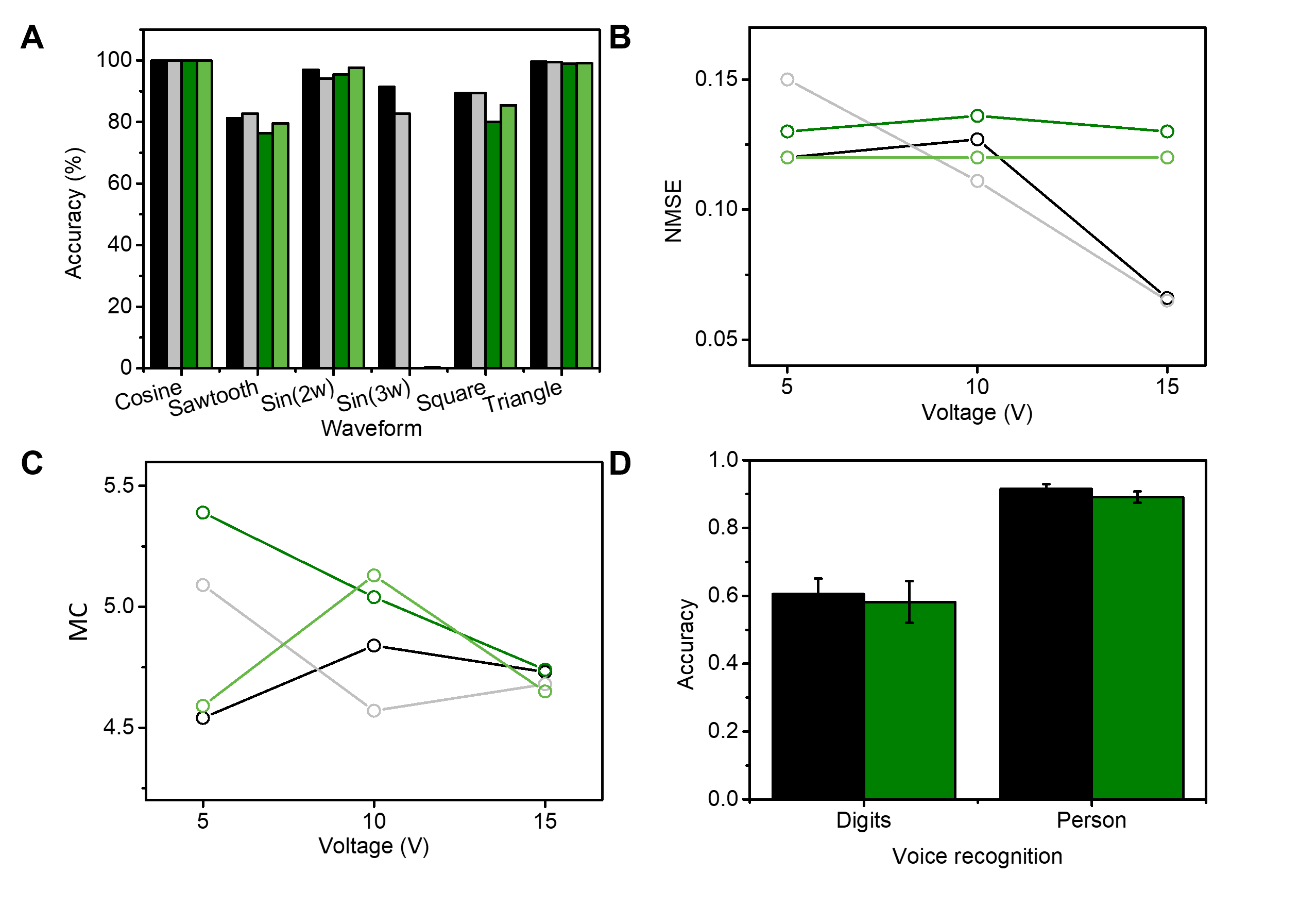


**Figure S12.** Effect of electrical poling on PRC tasks. (A-C) Comparison of (A) prediction accuracy for all waveforms (B) NMSE of NARMA2 task, and (C) MC values across YMO_⊥,s_ (black), YMO_⊥,o_ (grey), YMO_⊥,s_-poled (green), and YMO_⊥,o_-poled (light green). (D) Comparison of average prediction accuracy for voice recognition between YMO_⊥_ (black) and YMO_⊥_-poled (green). Error bar: standard error, N = 6 for digits and 10 for person. Input amplitudes: ±15 V for (A) and (D).

**Table S3.** Comparison of in-materio PRC performance across different device configurations.

| Device configuration | Nonlinearity | Prediction performance | | | Power consumption (µW) |
| --- | --- | --- | --- | --- | --- |
|  |  | WG (Ave. Acc., N = 6) | MC | NARMA2  (NMSE) |  |
| YMO_⊥,s_ | 1.0-1.7 | (94±3) % | 4.73 | 0.066 | 1.76 |
| YMO_⊥,o_ | 1.0-1.9 | (94±3) % | 4.68 | 0.065 | 2.00 |
| YMO_//,s_ | 1.3-1.9 | (91±3) % | 4.81 | 0.098 | 2.19 |
| YMO_//,o_ | 1.0-1.8 | (93±3) % | 4.48 | 0.080 | 1.12 |

Ave. Acc.: average accuracy.

**Table S4.** Comparison of in-materio PRC performance with reported materials and technologies.

| Material | Accuracy | | | | Power consumption | Practical applicability | Ref. |
| --- | --- | --- | --- | --- | --- | --- | --- |
|  | Waveform generation  (Ave. Acc.) | MC | NARMA2  (NMSE) | Voice recognition  (Accuracy) |  |  |  |
| Ti/TiO_x_  /Pd dynamic memristor | - | - | - | - | ~ 22 µW | √√√ Thermally stable | 9 |
| Ag-Ag_2_S NP memristor | 88.99% | 12.15 | 0.131 | - | ~ 13.81 µW | √√ Narrow operational temperature ranges | 12 |
| Ag_2_Se device | 85.85% | - | - | > 80% | 264 µW | √√ Unstable at high temperatures | 13 |
| Membrane SOA-based photonic RC system | - | - | - | - | 23 mW | √ Hard to be scalable | 28 |
| Ag/PVP memristive nanowire networks | - | - | - | - | 75 mW | √√  Narrow operational temperature ranges | 29 |
| SPAN OEND | 92.15% | 33.9 | - | 66% (ten digits)  60% (six speakers) | - | √ Thermal degradable | 10 |
| Ag_2_S island network | - | 3.9 | - | - | ~ 7.5 µW | √√ Thermally sensitive | 11 |
| Electrochemical reaction-based RC system | - | - | 0.0624 | - | - | √ Hard to be scalable and thermally sensitive | 14 |
| Organic electrochemical networks | - | - | - | - | 0.8 µW | √  Thermal degradable | 15 |
| YMnO_3_ (YMO_⊥_) | 94% | 4.70 | 0.065 | 75% (ten digits)  98% (six speakers) | 1.77 µW | √√√ Thermally stable up to 800°C | This work |

Note: the number of “√” indicates the practical applicability. The more “√”, the better practical applicability.
